# Peptide Amphiphilic-Based Supramolecular Structures with Anti-HIV-1 Activity

**DOI:** 10.1021/acs.bioconjchem.1c00292

**Published:** 2021-07-13

**Authors:** Maria J. Gómara, Ramon Pons, Carolina Herrera, Paul Ziprin, Isabel Haro

**Affiliations:** † Unit of Synthesis and Biomedical Applications of Peptides, Institute of Advanced Chemistry of Catalonia (IQAC−CSIC), Jordi Girona, 18-26 08034 Barcelona, Spain; ‡ Physical Chemistry of Surfactant Systems, Institute of Advanced Chemistry of Catalonia (IQAC−CSIC), Jordi Girona, 18-26 08034 Barcelona, Spain; § Department of Medicine, Imperial College London, London W2 1PG, United Kingdom; # Department of Surgery and Cancer, St. Mary’s Hospital, Imperial College London, London W2 1PG, United Kingdom

## Abstract

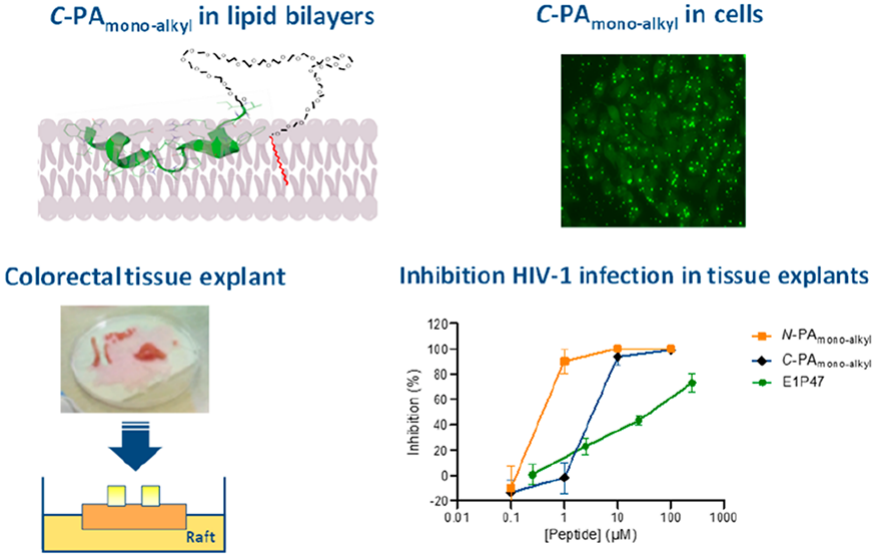

In a previous work, we defined a novel HIV-1 fusion inhibitor peptide (E1P47) with a broad spectrum of activity against viruses from different clades, subtypes, and tropisms. With the aim to enhance its efficacy, in the present work we address the design and synthesis of several peptide amphiphiles (PAs) based on the E1P47 peptide sequence to target the lipid rafts of the cell membrane where the cell–cell fusion process takes place. We report the synthesis of novel PAs having a hydrophobic moiety covalently attached to the peptide sequence through a hydrophilic spacer of polyethylene glycol. Characterization of self-assembly in condensed phase and aqueous solution as well as their interaction with model membranes was analyzed by several biophysical methods. Our results demonstrated that the length of the spacer of polyethylene glycol, the position of the peptide conjugation as well as the type of the hydrophobic residue determine the antiviral activity of the construct. Peptide amphiphiles with one alkyl tail either in C-terminus (*C*-PA_monoalkyl_) or in N-terminus (*N*-PA_monoalkyl_) showed the highest anti-HIV-1 activities in the cellular model of TZM-bl cells or in a preclinical model of the human mucosal tissue explants.

## Introduction

Fusion/entry inhibitors are considered promising antiviral agents because they can prevent the transmission of enveloped viruses by inhibiting the viral entry into the host cell.^1^ Controlling the initial event of viral transmission is the best way to prevent its dissemination.^2^ Given that its mechanism of action focuses on the stage prior to the entry of the virus into the cell, it is considered that this type of inhibitor may have a prophylactic potential similar to that provided by vaccine-induced immunity.

The mechanism of viral fusion with the host cell membrane is similar in most viruses that cause emerging or reemerging infectious diseases and is mediated by fusion glycoproteins.^3^ Despite structural differences between fusion proteins, they fold into a similar hairpin conformation and share common characteristics, thus constituting a therapeutic target for the development of fusion/entry inhibitors with broad spectrum activity.

Among this class of inhibitors, fusion inhibitor (FI) peptides deserve special attention because, unlike organic molecules of low molecular weight, they can mimic the structure of the protein domains involved in the process of fusion and are large enough to specifically inhibit protein–protein interactions. Due to their high specificity and ability to access hard-to-reach targets, peptides can offer the combined advantages of small molecules and large ones such as biologics.^4,5^ In addition to their high specificity, peptide-based viral inhibitors are considered to be safe therapeutic agents for human application *in vivo* and therefore might have a better acceptability rate than current antiviral drugs.^6^

In the last 30 years, HIV has been one of the most studied enveloped viruses, and it has been taken as a study model in the development of peptide-based fusion/entry inhibitors.^7,8^ In fact, the 36-mer peptide, T20 or Enfuvirtide, has been the first peptide-based entry inhibitor approved by the FDA for clinical use in HIV-infected patients.^9^ In contrast to the already mentioned advantages offered by peptide-based entry inhibitors, such as high specificity and safety, one of the major drawbacks of this type of molecules is its susceptibility to proteolytic degradation, thus having shorter half-life *in vivo* as well as lack of oral bioavailability. With this in mind, optimization of both sequence and structure of peptide-based inhibitors has been carried out in order to prolong their half-life, increase their antiviral activity, and improve druggability. One of these strategies is based on the modification of entry inhibitor peptides with hydrophobic residues to facilitate peptide anchoring to the viral target cell membrane.^10,11^ The conjugation of entry inhibitor peptides with saturated fatty acids or cholesterol allows obtaining lipophilic peptides capable of being located in lipid rafts. These are rigid gel-phase domains of the cell membrane where cell receptors and coreceptors are recognized by the HIV-1 envelope glycoprotein.^12^ Thus, targeted delivery of inhibitory peptides to these membrane regions constitutes a strategy to enhance their efficacy. In addition to the preclinical evaluation of cholesterol-derivatized peptide inhibitor C34 as a potential microbicide candidate,^13^ recent studies have shown that lipid derivatization of T20 FI and new generations of optimized sequences greatly improve antiviral potency and *in vivo* stability.^14−16^

In this study, we address the design of lipopeptides based on a peptide sequence which was defined by our group as an HIV-1 entry inhibitor. An 18-mer synthetic peptide, namely, E1P47, has demonstrated broad-spectrum activity against HIV viruses from different clades, subtypes, and tropisms. Biochemical and biophysical assays demonstrated that this peptide sequence interacts with the highly conserved N-terminal region of the HIV-1 gp41, which is the fusion domain, essential for viral entry.^17^ This interaction was subsequently confirmed by different NMR experiments in dodecylphosphocholine micelles (i.e., peptide–peptide titration, diffusion NMR spectroscopy, and addition of paramagnetic relaxation agents) showing the first evidence that the interaction between the inhibitory peptide and its viral target takes place at the membrane level.^18^

Considering that the lead peptide E1P47 targets a different region within the HIV-1 gp41 protein than the prototype T20 and C34 FIs, the design of lipopeptides based on the E1P47 peptide sequence could be of great interest to increase the repertoire of antiviral compounds. However, due to the high proportion of aromatic and hydrophobic amino acid residues in E1P47, conjugation of the peptide with a lipophilic group is expected to be troublesome in terms of solubility in physiological media. To overcome this drawback, we propose the design of novel peptide amphiphiles (PAs) which can provide nanostructures with higher bioavailability in physiological conditions. Classically, PAs consist of a biofunctional hydrophilic peptide segment bound to a hydrophobic alkyl lipid-like tail to create molecules with distinct hydrophobic and hydrophilic ends.^19^ PA self-assembly is promoted by the hydrophobic collapse of alkyl groups in aqueous solution to provide a hydrophobic core that leads to the formation of highly ordered three-dimensional nanostructures such as micelles, vesicles, and fibrillar structures (nanotubes or fibrils). PAs show increased amphiphilicity compared to natural peptides, as well as greater compatibility with the phospholipid bilayer.^20,21^ We report the synthesis of novel PAs having a hydrophobic moiety covalently attached to the amphiphilic peptide sequence through a hydrophilic spacer of polyethylene glycol (PEG). The ethoxyl groups of PEG confer high solubility to the PAs in their monomeric form without altering the net charge, and it is expected that they contribute to reduce recognition by the host response system as well as enzymatic degradation.^22^ In addition, it has been described that PEG may allow monomers to obtain a large headgroup to a relatively short and stiff tail. Mono- and dialkyl chains as well as cholesterol have been incorporated within the peptide structure as hydrophobic moieties taking into account their high affinity to the highly ordered domains of the host cellular membrane where the virus fusion process takes place.

Characterization of PA self-assembly in condensed phase and aqueous solution and their interaction with model membranes was analyzed by several biophysical methods. Finally, antiviral activities as well as the membrane mediated disassembly of PAs were also studied in cellular and tissue explant models with the aim of determining if the structural and membrane-mediated functional features might lead to increased antiviral potency.

## Results

In the present work, trying to improve the targeting to the viral membrane of the FI E1P47 peptide and consequently its antiviral activity, mono- and dialkyl groups (*N*-succinyl-octadecylamine and *N*-succinyl-dioctadecylamine) have been used as lipophilic tails. Moreover, taking into account that the HIV-1 envelope glycoprotein is located in lipid rafts during the process of viral fusion and that these subdomains of the plasma membrane contain 3- to 5-fold the amount of cholesterol found in the surrounding bilayer,^23^ we have also obtained E1P47 derivatives containing cholesterol within its primary structure. Besides, due to the intrinsic amphiphilicity of the E1P47 peptide, we have considered appropriate the incorporation of a PEG linker as the real hydrophilic moiety between the peptide sequence and the hydrophobic tails to obtain the novel PAs.

### Effect of the Length of the PEG Linker in the Design of Amphiphilic Peptides with Antiviral Activity

**Figure 1:**
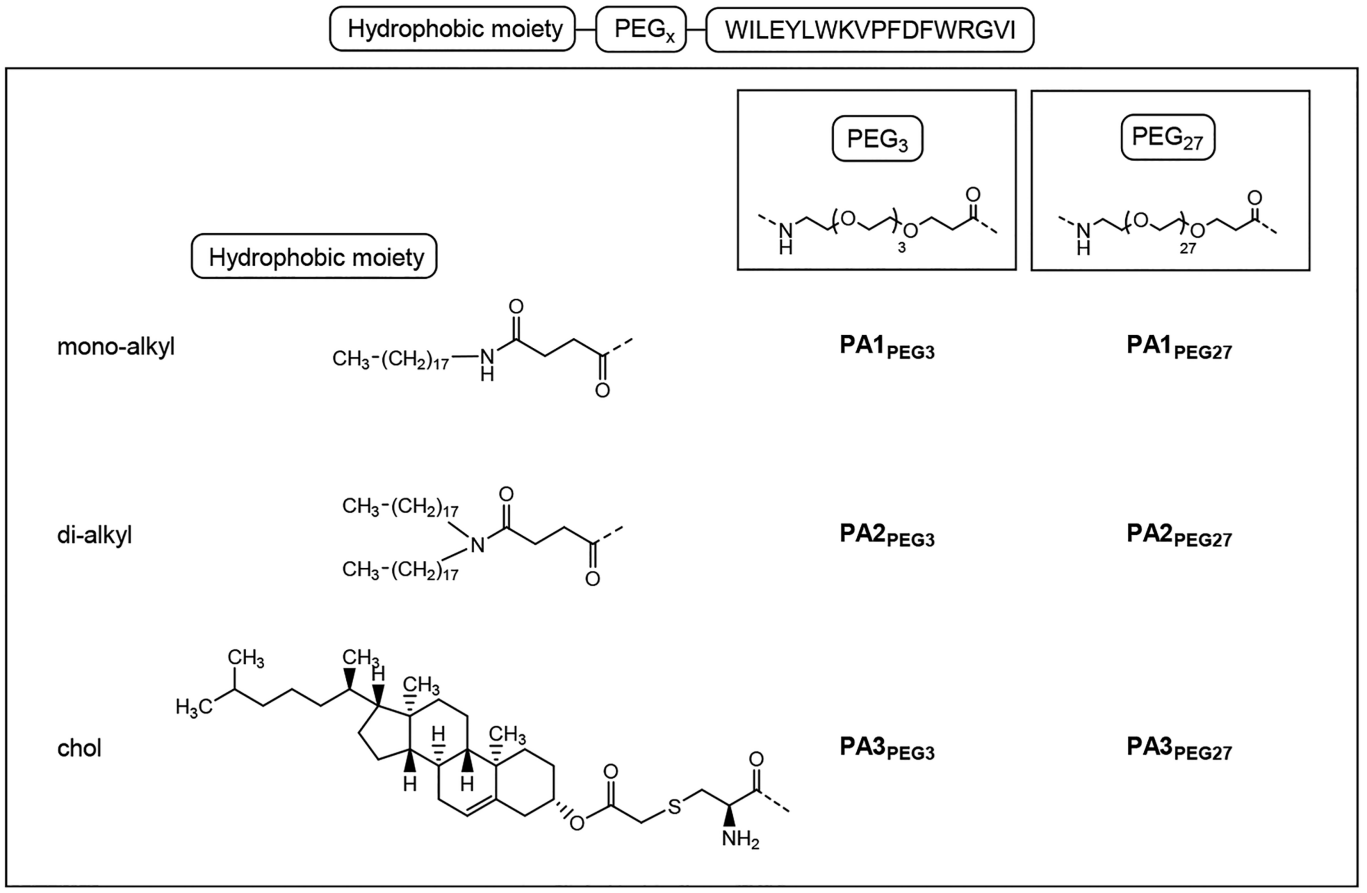
Scheme of synthesized *N*-peptide amphiphiles.

Taking into account that the PEG length could affect the peptide location after the self-assembly process of the supramolecular structure and, consequently, its location in the viral membrane, we first analyzed its impact on the antiviral activity of the PAs. With this aim, 2 different PEG spacers were chosen: a short one composed of 3 units of ethylene oxide (PEG_3_) and a longer polyoxyethylene one composed of 27 units (PEG_27_). On the other hand, and based on our previous results where we demonstrated that the lipophilic conjugation on *N*-terminus was able to enhance the antiviral activity of the FI peptides,^24^ we selected the *N*-terminal end of the peptide sequence to obtain the 6 PAs illustrated in Figure 1.

The synthesis of the *N*-PAs was carried out on the solid phase following the orthogonal protection strategy Fmoc/tBu. The mono- and dialkyl moieties were previously synthesized to obtain the *N*-succinyl-alkyl derivatives. The Fmoc-PEG_*X*_-COOH derivatives (PEG_3_ or PEG_27_) as well as *N*-succinyl-octadecylamine or *N*-succinyl-dioctadecylamine were stepwise coupled on the solid phase to the N-terminus of the E1P47 peptide sequence to obtain PA1_PEG3_, PA2_PEG3_, PA1_PEG27_, and PA2_PEG27_, respectively. To obtain PA3_PEG3_ and PA3_PEG27_, the cholesterol derivative, cholest-5-en-3-yl bromoacetate was previously synthesized from cholesterol and coupled in solution to the N-cysteinyl-PEG-peptide. The PAs were purified by flash chromatography and characterized by ES-MS (Experimental procedures and Figure S1 of Supporting Information).

The antiviral activity of the PAs was first assessed in TZM-bl cells. Table 1 shows the sensitivity of HIV-1_BaL_ to PAs expressed as IC_50_ and IC_95_ values calculated from the sigmoidal dose–response curve of inhibitory activity obtained from the titration of each peptide against this virus. PEG_27_ containing PAs showed lower IC_50_ values demonstrating higher antiviral potency than PAs with the PEG_3_ linker. Compared to the parent peptide (E1P47), the PA_PEG27_ conjugates showed IC_50_ values about 1 order of magnitude lower. The dose–response curves for PA1_PEG3_, PA2_PEG3_, PA2_PEG27_, PA3_PEG3_, and PA3_PEG27_ did not reach 100% of inhibition at the range of concentrations tested, so it was not possible to obtain IC_95_ values (Figure S2, Supporting Information). PA1_PEG27_ was capable of fully inhibiting infection of TZM-bl cells by HIV-1_BaL_ within the range of concentrations tested with a submicromolar IC_50_ value.

**Table 1:** Sensitivity of HIV-1_BaL_ to PAs in TZM-bl Cells

		monoalkyl (PA1)		dialkyl (PA2)		Chol (PA3)	
Inhibitory concentration^a^	E1P47	PEG_3_	PEG_27_	PEG_3_	PEG_27_	PEG_3_	PEG_27_
IC_50_ (μM)	7.26	0.23	0.20	N/A	0.15	2.01	0.12
	(2.22)	(0.23)	(0.04)		(0.04)	(1.29)	(0.07)
IC_95_ (μM)	28.21	N/A	5.87	N/A	N/A	N/A	N/A
	(2.01)		(0.72)				

a The IC values shown are the means (SEM) derived from the triplicates for each condition performed.

Thus, and according to the antiviral results, PEG_27_ was chosen as the PEG spacer for the further design of novel PAs containing the HIV-1 FI peptide as will be described in the next section.

To elucidate if the antiviral activity of PAs with different lengths could be related to the peptide location after self-aggregation of PAs in aqueous solution, we studied the exposure of the peptide on the self-assembled supramolecular structure.

**Figure 2:**
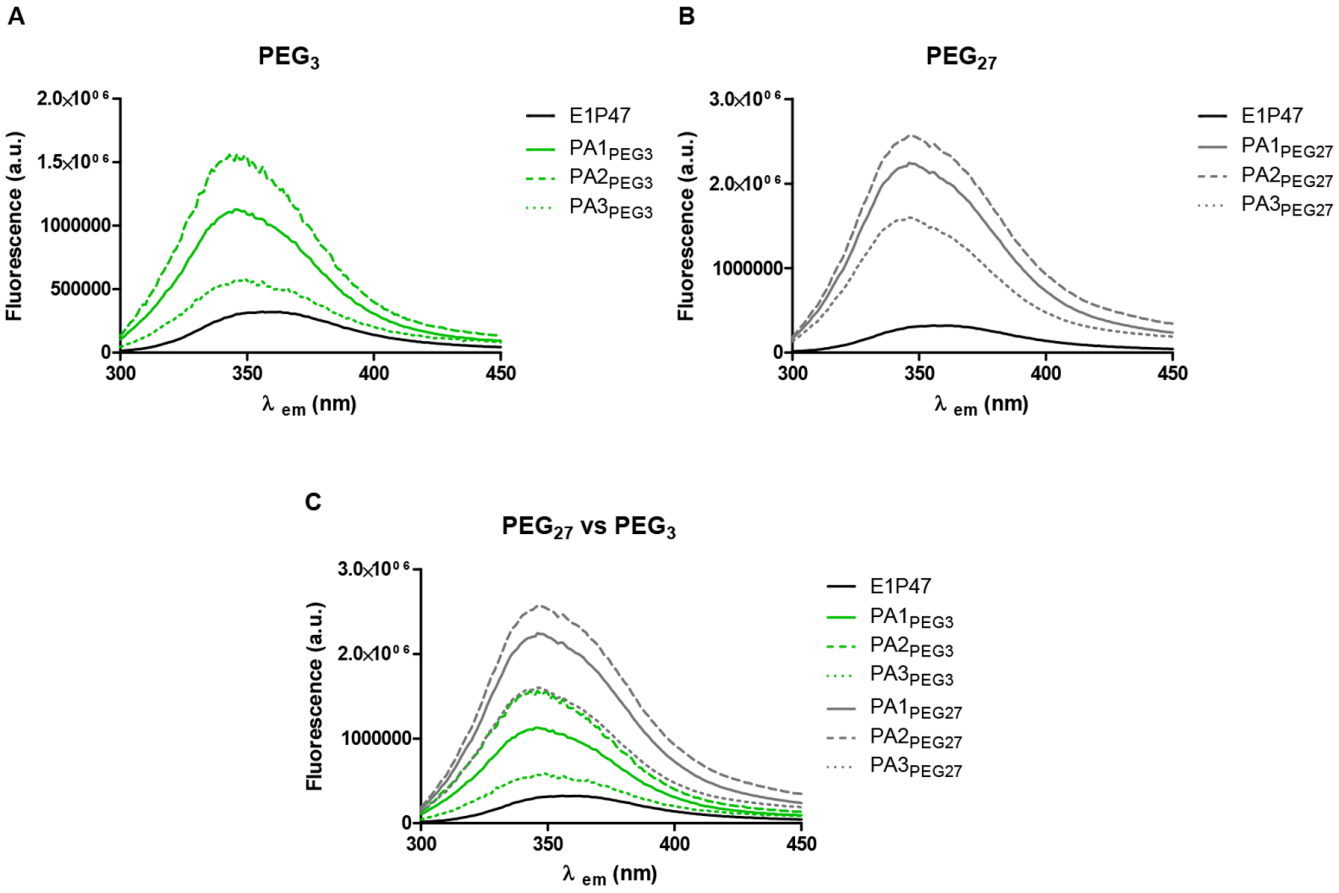
Fluorescence emission spectra of *N*-PAs in HEPES buffer (5.0 × 10^–6^ M). Comparison of the emission fluorescence depending on the length of the PEG linker.

As widely described, the intrinsic fluorescence emission of tryptophan can be used to probe the changes that occur in the tertiary conformation of oligomeric species formed during the peptide and protein aggregation process.^25−28^ Hence, we compared the fluorescence properties of the tryptophan residues in the PAs with those of the parent peptide in water. As shown in Figure 2, the emission spectrum of peptide E1P47 in water showed a maximum centered at 356 nm indicating the hydrophilic environment of the W residues. However, the incorporation of PEG and hydrophobic groups to the peptide sequence led to conjugates that showed emission spectra of higher intensity (Figure 2A and B). Furthermore, the maximum emission wavelength was around 10 nm blue-shifted compared to the maximum emission of the E1P47 peptide, indicating that the tryptophan residues in the PAs are located in a more hydrophobic environment.^29^ As shown in Figure 2C, the intensity of the emission spectra of the PAs clearly depended on the PEG length. Conjugates containing the longest PEG showed higher fluorescence intensity indicating that their tryptophan residues were closer to the nonpolar environment created by the hydrophobic moiety of the PA. These results led us to hypothesize that PEG_27_ derivatives might be forming an aqueous exposed loop that would facilitate a close contact between the peptide and the hydrophobic moiety within those PAs, whereas PEG_3_ seemed to be not long enough to allow it. This suggests that the PEG_27_ driven self-assembled structures might allow a more effective way of incorporating the FI peptide to the cell membrane which is where inhibitory activity takes place.

### Effect of the Position of Conjugation within the Peptide Sequence in the Design of Amphiphilic Peptides with Antiviral Activity

We then analyzed the position of the conjugation within the peptide sequence. First, we characterized the self-aggregation of the parent E1P47 peptide sequence in aqueous media as well as its interaction with model membranes. E1P47 peptide has three tryptophan residues on its sequence: at the *N*-terminus (W^1^), at the middle (W^7^), and at the *C*-terminal end (W^14^). Based on this, we synthesized three peptide analogues in which two tryptophan residues were replaced by the aromatic phenylalanine analogue in order to study the local environment of each particular tryptophan. Based on the emission spectrum of W^14^-E1P47, the maximum emission wavelength (λ_max_) was centered at 339 nm indicating that the tryptophan located at the *C*-terminal region was buried and not exposed to the aqueous media. Regarding the emission spectra of W^1^-E1P47 and W^7^-E1P47 peptides, W^7^ displayed a broad red-shifted fluorescence at 355 nm relative to W^1^ whose maximum emission was centered at 345 nm (Figure S3A, Supporting Information). These results indicated that the E1P47 peptide tends to adopt an orientation in aqueous media where the C-terminal end is buried and the central part of the peptide close to W^7^ is exposed to the aqueous media. To characterize the local disposition of each particular tryptophan in a hydrophobic environment, we then studied the intrinsic fluorescence of the peptides in the presence of POPC liposomes at a peptide/lipid ratio of 1/100 (Figure S3B Supporting Information, dashed lines). Fluorescence intensity of W^14^-E1P47 did not increase after adding liposomes, thus indicating that the *C*-terminal end of the peptide remained buried. On the contrary, upon addition of liposomes the fluorescence intensity of W^1^-E1P47 increased around 2-fold showing that the *N*-terminal end of the peptide interacted with the membrane. The fluorescence emission of W^7^-E1P47 was 15 nm blue-shifted and increased the intensity of the maximum, thus demonstrating that the central part of the peptide also interacted with the hydrophobic membrane.

E1P47 tended to form aggregates in aqueous media where the *C*-terminal end seemed to drive the molecular packaging. The overall results agreed well with the previous structural characterization of the peptide by NMR in the presence of micelles. The experimental information derived from the NOE measurements was used as distance restrains to obtain by molecular dynamics simulations the three-dimensional helix–turn–helix peptide structure.^18^

Intrinsic fluorescence analysis of E1P47 demonstrated the different exposure of the tryptophan residues to the aqueous medium according to their position in the peptide sequence. Therefore, in addition to the *N*-terminal conjugates shown in Figure 1, we also synthesized novel conjugates at the *C*-terminal end and at the K^8^ position, this last being located next to the W^7^ residue (Figure S4, Supporting Information). Since the *C*-terminal end of the peptide sequence was buried and the central loop was exposed to the aqueous medium, we expected that the position of the conjugation would affect the PA self-assembly process rendering a different arrangement of the peptide sequence in the resulting supramolecular structure.

**Figure 3:**
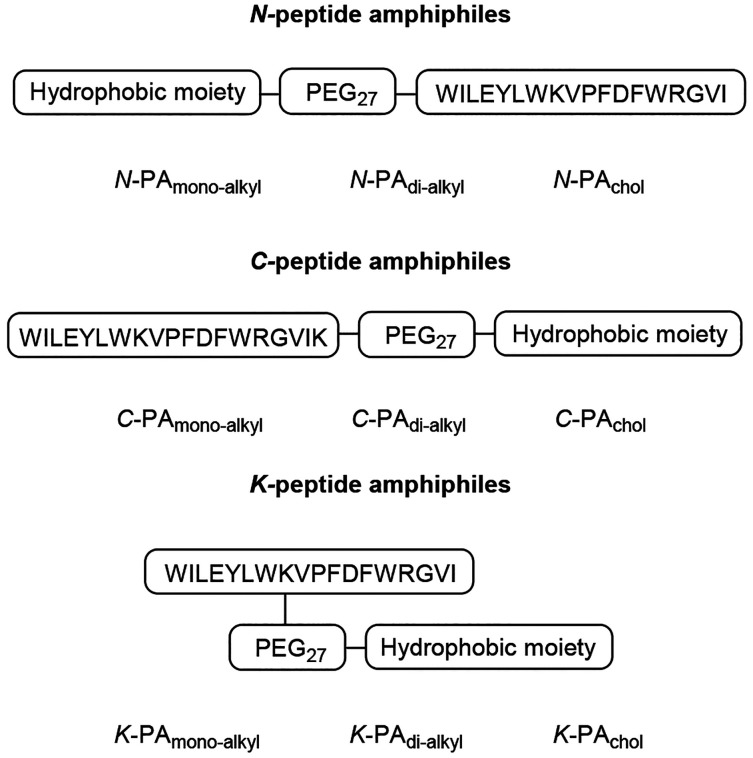
Schematic representation of the synthesized *N-*, *C-*, and *K-*peptide amphiphiles.

A schematic representation of the novel *N-, C-*, and *K-*PAs is shown in Figure 3. The syntheses were carried out similarly to that performed previously for *N*-derivatized PAs but with the introduction of an orthogonal lysine derivative at the *C*-terminus (for *C*-PA_monoalkyl_, *C*-PA_dialkyl_, and *C*-PA_chol_ derivatives) or in the K^8^ position (for *K*-PA_monoalkyl_, *K*-PA_dialkyl_, and *K*-PA_chol_ derivatives). The selective deprotection of the lysine derivative allowed the subsequent derivatization of the peptidyl-resin on the solid phase. The PAs were conveniently purified and characterized by ES-MS (Experimental procedures of Supporting Information).

### Analysis of Self-Organization of PAs

#### Critical Micellar Concentration Measurements

These compounds are amphiphilic in nature; hence, the surface tension as a function of the concentration in DMSO containing solutions was studied. All nine conjugates showed surface tension reduction down to values that could be considered as surfactants (values between 30 and 39 mN m^–1^; see Figure S5, Supporting Information). The form of the plots of surface tension as a function of concentration did not differ from those of classical surfactants. At low concentrations, the surface tension was close to that of pure water (DMSO at 0.5% did not change that value significantly) and progressively decreased to a stabilization point. This concentration is usually identified as the Critical Micellar Concentration (CMC); however, strictly speaking this identification is overstating,^30^ and the stabilization of surface tension rather marks the saturation of the surface by the added molecules. There was not a clear trend in the apparent CMC values. The range of values spanned from 7.1 ± 0.5 μM to 1.7 ± 0.2 μM, which were remarkably low; however, in general, the introduction of an octadecanoyl chain should have induced a drastic decrease of CMC (1 order of magnitude for the elongation of a single chain with four methylene groups as seen in dodecyl trimethyl ammonium bromide 14.5 mM compared with hexadecyl trimethyl ammonium bromide 0.8 mM,^31,32^ or a factor of 50 for incorporating a second decanoyl chain when comparing decyl trimethyl ammonium bromide CMC 66.8 mM with that of didecyl dimethyl ammonium bromide 1.4 mM).^33,34^ In the same direction, the surface tension–concentration plot of E1P47 looks very similar to the PAs curves, with CMC and limiting surface tension both in the higher end (6.5 μM and 38 mN m^–1^ respectively).

#### X-ray Scattering Measurements

We then attempted to obtain further details on the self-organization of the PAs by measuring the X-ray scattering in condensed phase in excess water.

With humidity (48 h incubation with excess water at 40 °C), *C*-derivatives showed the presence of a lamellar-like ordering (see Figure S6), with characteristic distances of 4.7, 5.7, and 4.9 nm for *C*-PA_monoalkyl_, *C*-PA_dialkyl_, and *C*-PA_chol_, respectively (note that all spectra of cholesterol derivatives showed a small but significant peak around 1.6 nm^–1^, similar to that found for the precursor *cholest-5-en-3-yl bromoacetate)*. Unfortunately, the assignation was not univocal; only the 100 is clearly observed, and for the *C*-PA_monoalkyl_ the 300 reflection is also evident; the absence of 200 reflections in lamellar structures is frequent and implies symmetric or nearly symmetric electron density fluctuations. With the molecules being strongly asymmetric, this could be achieved in several ways. We considered three slabs connected by error functions. This model fairly agreed with the observed spectra of *C*-PA_monoalkyl_ (and hence with the single peak observed for *C*-PA_dialkyl_ and *C*-PA_chol_); both the electronic profile and the comparison of the experimental with fitted spectra are shown in the Supporting Information Figure S6, Supporting Information. According to these results, in this sample the peptide, polymer, and hydrocarbon domains would segregate.

*N*-PA_monoalkyl_, *N*-PA_dialkyl_, and *K*-PA_monoalkyl_ presented similar profiles with two bands around 0.6 and 1.4 nm^–1^. This was compatible with interacting globular structures of about 4–7 nm in radius (see fits and parameters in Figure S6 and Table S1, Supporting Information). These relatively undefined structures would agree with the view of small aggregation numbers for micelles of these molecules. In the *N*-PA_chol_ derivative, this was less defined as well as in *K*-PA_chol_. Changes were observed in these last two samples after one month at 40 °C (see Figure S6), with *K*-PA_dialkyl_ becoming compatible with vesicular structures of around 9 nm bilayer thickness, and *K*-PA_chol_ showing the formation of a lamellar liquid crystal with periodicity 4.8 nm (with an additional peak present in the cholesterol precursor).

### Interaction Analysis of PAs with Membranes

#### PAs’ Affinity for HeLa-Env Cell Membranes

The ability of PAs to interact with the cell membrane was evaluated by *in vitro* flow cytometry assay using HeLa-Env cells, which express the HIV-1 envelope protein. HeLa-Env cells were treated with fluorescent PAs, obtained by their coassembly with the fluorescent lipophilic derivative FAM-C_18_ (Figure S7, Supporting Information). Table 2 shows the membrane association percentages of the FAM-C_18_ trapped in the self-assembled PAs, which resulted from subtracting the intracellular fluorescence from the total fluorescence.

**Table 2:** Membrane Affinity of PAs Studied by Flow Cytometry and Trp Fluorescence Emission

		flow cytometry assay	binding assay
hydrophobic moiety	PA	% membrane associated-fluorescence	*K_x_*^a^
monoalkyl	*N*-PA_monoalkyl_	36	1.2 × 10^–6^
	*C*-PA_monoalkyl_	63	3.8 × 10^–6^
	*K*-PA_monoalkyl_	41	0.24 × 10^–6^
dialkyl	*N*-PA_dialkyl_	25	ND
	*C*-PA_dialkyl_	27	ND
	*K*-PA_dialkyl_	26	ND
chol	*N*-PA_chol_	32	ND
	*C*-PA_chol_	47	2.4 × 10^–6^
	*K*-PA_chol_	51	0.9 × 10^–6^

a *K_x_* values of apparent mole fraction partition coefficients determined from partitioning isotherms of PAs estimated from the fractional change in Trp fluorescence intensity upon addition of increasing amounts of liposomes.

This assay allowed us to compare the disassembly of the different PA aggregates when they interact with the cell membrane, since the fluorescent FAM was not covalently bound to the PA but coassembled through hydrophobic intermolecular interactions. As shown in Table 2, FAM-C_18_ trapped in dialkyl conjugates demonstrated the lowest percentages of fluorescence associated with the membrane, indicating that these PAs aggregates were tightly packed, and the disassembly and subsequent association to the cell membrane was less effective. On the contrary, FAM-C_18_ trapped in the monoalkyl conjugates showed higher % of membrane associated fluorescence. Particularly, the fluorescent lipophilic derivative trapped in *C*-PA_monoalkyl_ showed the highest percentage of membrane binding (63%).

**Figure 4:**
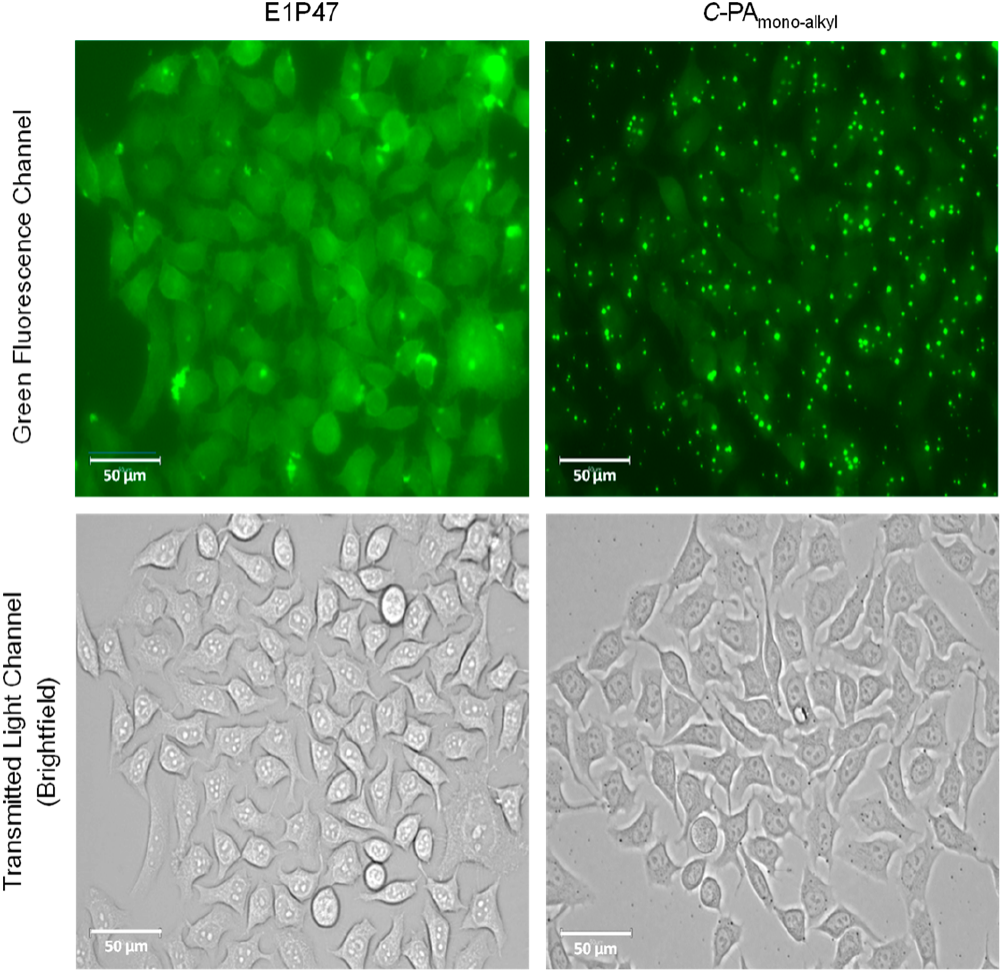
Captured images in EVOS M7000 fluorescence microscope with green fluorescence and transmitted light channels on HeLa-Env cells treated with 6 μM of either *C*-PA_monoalkyl_ or unconjugated E1P47 both coassembled with FAM-C_18_ for 4 h.

In addition, these fluorescent coassembled structures enabled the use of optical microscopy to directly observe self-assembly of the peptide conjugates. As an example, Figure 4 shows captured images of HeLa-env monolayers treated with FAM-C18 trapped in either *C*-PA_monoalkyl_ or unconjugated E1P47. Comparatively, the pattern distribution for E1P47 and the monoalkyl conjugate were different in live cells. E1P47 revealed a more diffuse and uniform cell pattern distribution, whereas the PA showed a punctate distribution compatible with the formation of discrete nanostructures. The coassembly of the peptide-conjugate with a fluorophore-modified derivative provides a significant illustration of the formation of self-assembled nanostructures.

#### Study of PAs’ Interaction with POPC Model Membranes by Fluorescence Measurements

Membrane affinity of PAs aggregates was further studied through Trp fluorescence emission of PAs in the presence of model membranes (Figure S8, Supporting Information). Partitioning isotherms were estimated from the fractional change in Trp fluorescence intensity upon addition of increased amounts of POPC liposomes. Interestingly, it was only possible to estimate partitioning isotherms for the PAs that gave the higher percentages of membrane association of the fluorescent derivative on the flow cytometry assay (Figure S9, Supporting Information). The apparent mole fraction partition coefficients determined are shown in Table 2. The results from the binding assay with model membranes agreed with the flow cytometry assay data using HeLa-Env cells, with the binding percentages of the fluorescent lipophilic derivative determined in the flow cytometry assay being similar to the binding percentages of the respective disassembled PA.

We could not obtain the apparent mole fraction partition coefficients for dialkyl PAs which gave the lowest percentages of membrane association on the flow cytometry assay (Table 2). On the contrary, the monoalkyl conjugate in *C*-terminus (*C-*PA_monoalkyl_) gave the highest apparent partition coefficient (*K*_*X*_ = 3.8 × 10^6^) and the highest percentage of membrane association, as previously indicated.

#### Study of PAs’ Interaction with POPC Model Membranes by Small Angle X-ray Scattering (SAXS) Measurements

Further insight into the interaction of PAs with membranes was obtained by measuring the SAXS spectra in a model membrane of POPC in the presence and in the absence of PAs.

**Figure 5:**
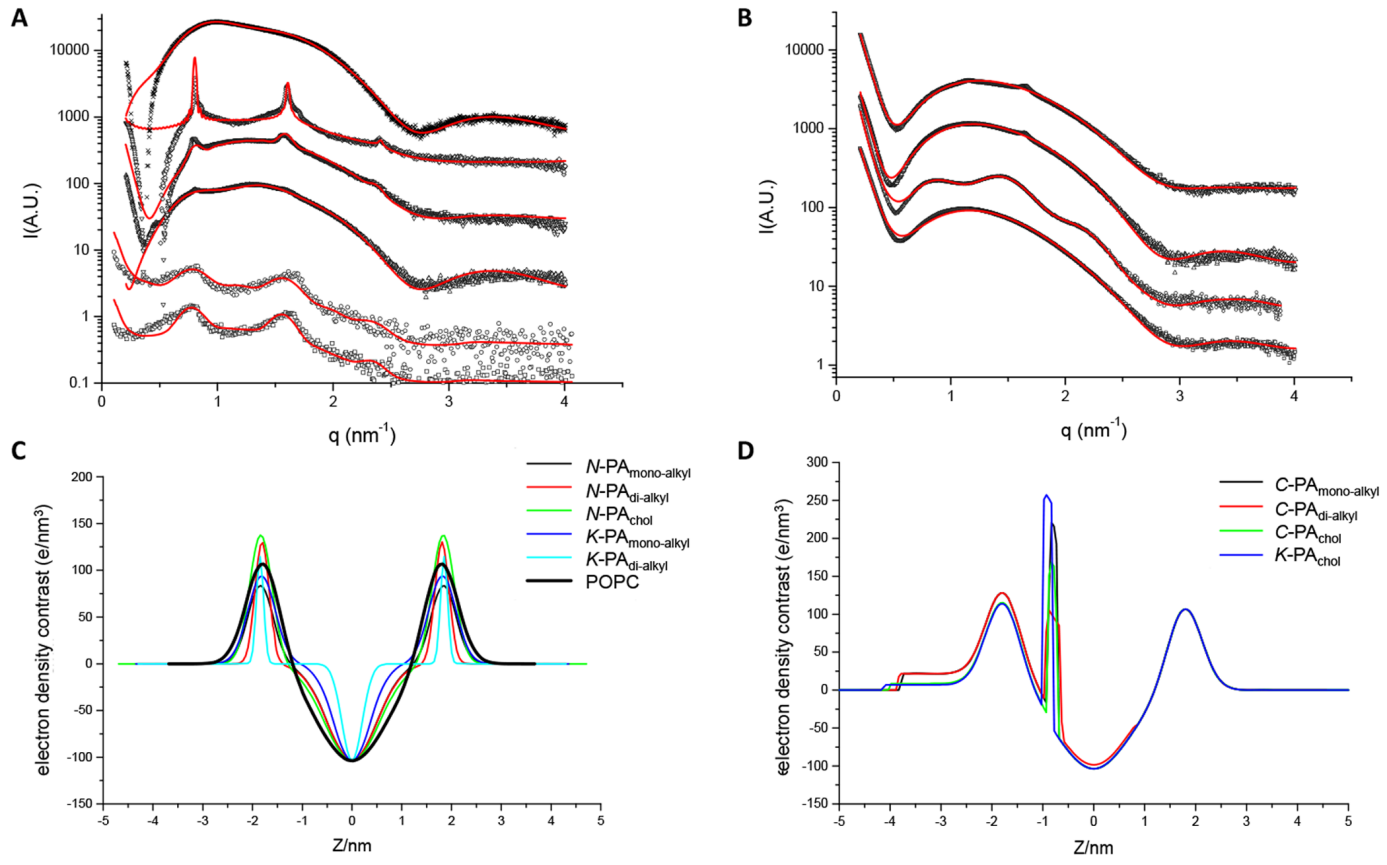
(A) SAXS intensity as a function of dispersion vector modulus **q** for samples of 20 mM (in 0.5% DMSO); POPC with 2 mM *N*-PA_monoalkyl_ (squares), *N*-PA_dialkyl_ (circles), *N*-PA_chol_ (up triangles), *K*-PA_monoalkyl_ (down triangles), and *K*-PA_dialkyl_ (diamonds) and in the absence of PAs crosses (respective curves in the order from bottom to top), together with the best fits as lines. Note that *N*-PA_monoalkyl_ and *N*-PA_dialkyl_ were measured in an in-house instrument and the rest using synchrotron. (B) SAXS intensity as a function of dispersion vector modulus **q** for samples of 20 mM (in 0.5% DMSO); POPC with 2 mM *C*-PA_monoalkyl_ (squares), *C*-PA_dialkyl_ (circles), *C*-PA_chol_ (up triangles), and *K*-PA_chol_ (down triangles) (bottom to top), together with the best fits as lines. Spectra are displaced in the *y*-axis for clarity. (C) Electron density contrast as a function of the distance to the center of the bilayer for: *N*-PA_monoalkyl_, *N*-PA_dialkyl_, *N*-PA_chol_, *K*-PA_monoalkyl_, and *K*-PA_dialkyl_ and in the absence of PAs. (D) Electron density contrast as a function of the distance to the center of the bilayer for *C*-PA_monoalkyl_, *C*-PA_dialkyl_, *C*-PA_chol_, and *K*-PA_chol_.

In Figure 5A,B, we show the spectra corresponding to POPC 20 mM and POPC 20 mM + 2 mM PAs (all of them in the presence of 0.5% DMSO). The band centered at 1.2 nm^–1^ and the secondary band at 3.2 nm^–1^ were typical of unilamellar vesicles.^35^ The figure also shows the fit of a Gaussian model (see the details in the Experimental Procedures), and the obtained parameters agreed with those in the literature for POPC multilamellar vesicles,^36^ which are also similar to POPC/POPG 95/5 unilamellar vesicles.^37^

The incorporation of PAs had some distinct consequences. The first and more obvious one is the distinction between stable and unstable systems. *N*-Terminal alkyl modified peptides (*N*-PA_monoalkyl_, *N*-PA_dialkyl_) precipitated rather fast and made compact flocks (this prevented measuring those two samples at the synchrotron and were measured at a later time in our in-house instrument). The rest of the samples did not visibly separate, but they had different aspect; while *C*-terminal hydrophobically modified peptides (*C*-PA_monoalkyl_, *C*-PA_dialkyl_, and *C*-PA_chol_) as well as *K*-PA_chol_ were bluish, the peptides that were modified with alkyl tails in the K^8^ position (*K*-PA_monoalkyl_ and *K*-PA_dialkyl_) as well as *N*-PA_chol_ were milky.

When examined by SAXS, widely different behavior was observed. *N*-PA_monoalkyl_, *N*-PA_dialkyl_, and *K*-PA_dialkyl_ spectra showed strong peaks (note that our in-house instrument has a strong smearing effect, particularly noticeable in the peaks). The rest of the samples showed bands similar to those of POPC (Figure 5A,B). The samples that show strong multilamellarity (N-PA_monoalkyl_, N-PA_dialkyl_ K-PA_monoalkyl_, and K-PA_dialkyl_) exhibit peaks corresponding to a repeating distance of 7.8 ± 0.1 nm, sensibly larger than that of POPC alone in multilamellar samples 6.6 nm at 2 °C or 6.4 nm at 50 °C^36^ or the signs of multilamellarity found here (6.7 nm). This should correspond then to the effect of partial swelling due to the peptide.

We fitted all the samples to models based on Gaussian descriptions of the polar head-groups of the phospholipid and the methyl end groups (shown as lines in Figure 5C–D and fitting parameters given in Table S2, Supporting Information). All the N-terminal modified peptides and those peptides that were modified with the alkyl tail (mono and dialkyl) in the K^8^ position were well fitted with this model. Some of them corresponded to correlated bilayers, notably *N*-PA_monoalkyl_, *N*-PA_dialkyl_, and *K*-PA_dialkyl_ which presented strong repetition peaks, with *K*-PA_monoalkyl_ showing a band of uncorrelated bilayers superimposed to two clear and narrow correlation peaks and *N*-PA_chol_ corresponding mostly to uncorrelated bilayers. The corresponding electronic density profiles are shown in Figure 5C–D.

We observed that the profiles were similar, with a dip at the center of the bilayer (corresponding to the methyl and methylene groups of the phospholipids) and two positive bands corresponding to the high-electronic-density headgroups of the phospholipid. However, it was unclear whether the PAs were present in the bilayer. The position of the headgroups was not sensitive to the presence of PAs with the main effect being a narrowing to the width of both the headgroups and the dip at the center of the bilayer. This narrowing may be concomitant with an increase in the order of the bilayer, also reflected in the formation of multilayers.

The rest of PAs (*C*-terminal derivatized peptides and *K*-PA_chol_) could not be sensibly fitted to the model and needed additional parameters. Because the minimum at the left of the main band was largely suppressed, the obvious reason was the asymmetry of the electron density profile.^38,39^ Of the many options, we adopted that of perturbing the symmetric model (and using the same parameters as for POPC) by the addition of three slabs representing the three blocks of the structure of PAs, which was consistent with the preparation procedure of the mixed systems. Using those three slabs, we only stipulated that those three slabs be consecutive, but we left free their lengths and electron density. Thanks to the defined chemical composition, we discarded electron density profiles incompatible with this knowledge. First, we conducted a grid search strategy, and with the best results, we further minimized. We found that there was a large slab with slightly above solvent electronic density, a narrow slab with high electron density close to the hydrophilic–hydrophobic interface of the bilayer, and a third slab in the hydrophobic domain that tended to be negligible (fit shown in Figure 5C–D as lines and corresponding fitting parameters given in Table S3, Supporting Information).

The fits were good, and they showed signs of multilamellarity for *K*-PA_chol_ (two small and defined peaks on the bilayer band representing less than 10% of the signal) and *C*-PA_dialkyl_ showing some signs of oligolamellarity. Samples *C*-PA_monoalkyl_ and *C*-PA_chol_ did not show any signs of correlation between bilayers.

From these results, it was clear that the vesicular structure was maintained in the samples that showed nonsymmetric bilayer structure, while aggregation occurred in the samples that showed symmetric bilayers. We should mention that, although the fits led to a narrow high electron density slab, the minimum with respect to this width and height was quite shallow with strong inverse correlation of width and height, keeping constant its product. This was consistent with the peptide blocks lying parallel to the bilayer surface at the hydrophilic side of the hydrophilic–hydrophobic interface. Based on the narrow width obtained by fitting, the peptide backbone of the peptide would be the responsible for this narrow high-electron-density change. There was no sign of the peptide inserting in the hydrophobic part of the bilayer, with the electronic density of this slab having no net contribution. The PEG block would be responsible for the small, but significant, increase of electronic density at distances up to 4 nm from the center of the bilayer.

### Antiviral Activity of PAs

The antiviral activity of PAs was tested against HIV-1_BaL_ in TZM-bl cells. The cytotoxic effect of the compounds was also assessed (Table S4, Supporting Information).

**Figure 6:**
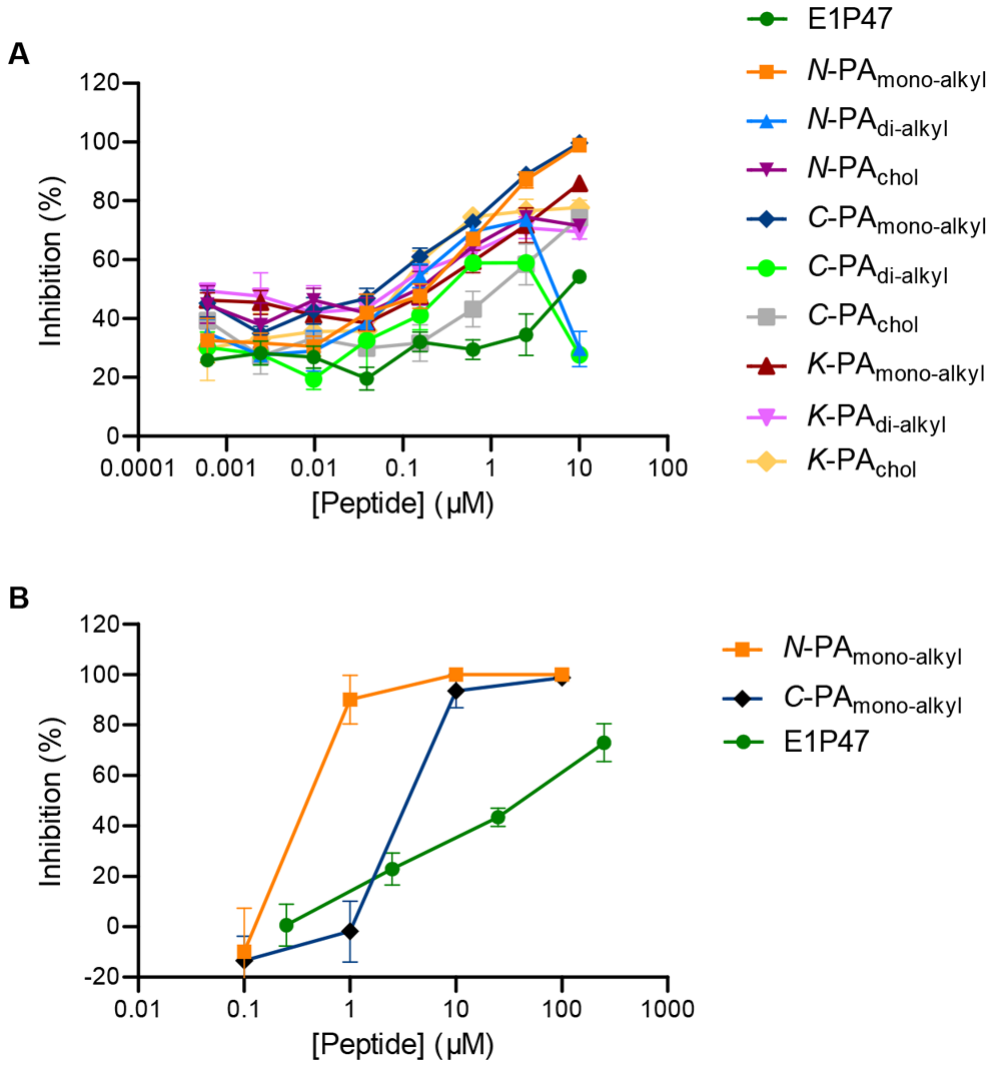
Inhibitory activity of E1P47 peptide derivatives against HIV-1_BaL_ infection. TZM-bl cells (A) and colorectal tissue explants (B) were treated for 1 h in the presence or absence of serial dilutions of peptides prior to addition of HIV-1_BaL_. Luciferase expression in TZM-bl cells (measured in relative light units) was determined after 48 h of culture, and the extent of inhibition by each drug was calculated. Tissue explants were washed four times with PBS after 2 h of incubation with virus and then transferred to gelfoam rafts. Explants were kept in culture for 15 days in the absence of peptide. The concentrations of p24 in the harvested supernatants were quantified by ELISA at day 15 of culture, and the extent of inhibition by each compound at each time point was calculated. The percentage of inhibition was normalized relative to the relative light units obtained for TZM-bl cells or to the p24 values obtained for tissue explants not exposed to virus (0% infectivity) and for cells or explants infected with virus in the absence of compound (100% infectivity). Data are the means (±SEM) from triplicates.

Table 3 shows the IC_50_ and IC_95_ values of all PAs. The dose–response curves of the derivatized PAs in the K^8^ position did not reach total inhibition in the range of the concentrations tested (Figure 6A), and it was not possible to obtain IC_95_ values.

**Table 3:** Sensitivity of HIV-1_BaL_ to PA_PEG27_ in TZM-bl Cells

	*N*-PA			*C*-PA			*K*-PA		
inhibitory conc.^a^	monoalkyl	dialkyl	chol	monoalkyl	dialkyl	chol	monoalkyl	dialkyl	chol
IC_50_ (μM)	0.20	0.15	0.12	0.08	N/A	1.71	0.37	0.05	0.09
	(0.04)	(0.04)	(0.07)	(0.01)		(0.72)	(0.17)	(0.04)	(0.01)
IC_95_ (μM)	5.87	N/A	N/A	5.60	N/A	N/A	N/A	N/A	N/A
	(0.72)			(0.93)					
SI (CC_50_/IC_50_)	85	-	-	250	-	-	-	-	-

a The IC values shown are the means (SEM) derived from the triplicates for each condition performed.

These results led us to suggest that lipophilic conjugation in the central loop of the peptide sequence did not provide an appropriate presentation of the peptide. Furthermore, *N*-PA_dialkyl_ and *C*-PA_dialkyl_, which contained two alkyl tails, lost their activity at the highest dose tested, demonstrating that, apart from the position of the lipophilic conjugation, the type of hydrophobic residue greatly affected the antiviral activity of the PAs. Similarly, *N*-PA_chol_ and *C*-PA_chol_ failed to achieve 100% inhibition, suggesting that cholesterol was not the hydrophobic moiety generating the most active PAs. Therefore, the best amphiphilic conjugates capable of inhibiting HIV-1_BaL_ infection of TZM-bl cells were the ones modified with only one alkyl tail either in the N- or in the C-terminus. Considering that *N*-PA_monoalkyl_ and *C*-PA_monoalkyl_ gave adjusted dose–response curves throughout the concentration range, the selective index (SI) values were calculated as the ratio between the 50% cytotoxic concentration (CC_50_) and the half-maximal inhibitory concentration (IC_50_). These monoalkyl conjugates, particularly the one derived at the C-terminal end (*C*-PA_monoalkyl_), demonstrated the highest antiviral potency with an IC_50_ value in the nanomolar concentration range and an SI of 250.

The inhibitory activity of *N*-PA_monoalkyl_ and *C*-PA_monoalkyl_ was then assessed in a preclinical model of human mucosal tissue explants. Colorectal explants were treated with peptides before and during viral exposure (3 h) as a “pulse” condition to mimic drug dosing immediately prior to intercourse and during exposure. A dose–response curve was observed for all peptides against HIV-1_BaL_ allowing us to calculate IC_50_ and IC_95_ values (Table 4, Figure 6B). *N*-PA_monoalkyl_ showed greater inhibitory activity than *C*-PA_monoalkyl_ in contrast to what we observed in TZM-bl cells.

**Table 4:** Inhibitory Activity of *N*-PA_mono-alkyl_ and *C*-PA_mono-alkyl_ in Colorectal Tissue Explants

inhibitory concentration^a^	*N*-PA_monoalkyl_	*C*-PA_monoalkyl_
IC_50_ (μM)	0.28 (0.03)	3.48 (0.44)
IC_95_ (μM)	2.53 (1.14)	24.29 (3.61)

a The IC values shown are the means (SEM) derived from the triplicates for each condition performed.

## Discussion

This study allowed us to define the structural characteristics that determined the PA self-assembly in aqueous solution, their membrane affinity, as well as their disassembly promoted by the presence of either model membranes or cellular membranes.

First, the size of the PEG as a hydrophilic linker between the amphiphilic peptide and the hydrophobic moiety must be long enough to allow the self-assembly of the resulting conjugate according to their amphiphilic properties so that the polyethylene glycol chain is exposed to the aqueous environment.

**Figure 7:**
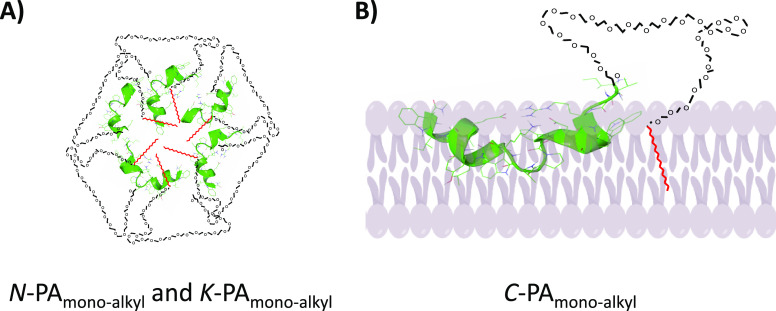
(A) Probable globular structure of *N-* and *K*-PAs (induce destabilization of lipid bilayers). (B) Probable disposition of *C*-PAs in the vesicle bilayer. Peptide is represented in green, hydrocarbon chain in red, and PEG moiety in black.

Second, the position of the conjugation of linkers determines the orientation of the peptide sequence on the conjugate. Based on previously reported structural studies as well as the fluorescence-based results presented in this work, the *C*-terminal domain of the peptide sequence E1P47 drives the molecular packaging. Therefore, the lipophilic derivatization at the *C*-terminal end favors the tendency of peptide aggregation so that the *N*-terminal part of the peptide is more exposed to interact with the membrane. In addition, the position of conjugated linkers showed a different self-organization of the PAs as measured by X-ray scattering in excess of water. Thus, *C*-terminal conjugates demonstrated the presence of a lamellar-like ordering, while *N*- and *K*- conjugates mainly present profiles compatible with globular structures that induce the aggregation of lipid bilayers as well as their destabilization. Furthermore, either C-derivatives do not form micelles or the monomer solubility is high enough to allow for the incorporation of the molecules to the bilayer in an effective way, which leads to the unsymmetrical bilayer observed by SAXS. The probable disposition of the PAs is shown in Figure 7. These observations are in line with our observations by fluorescence binding to the bilayer. The PAs with the lower proportion of bilayer associated fluorescence were those that produced unstable systems and symmetric bilayers (with the exception of *C*-PA_dialkyl_).

Third, the type of lipophilic residue mainly affects the nanostructure disassembly promoted by the presence of lipid membranes. Based on the results obtained from the flow cytometry assay tracking the fluorescent lipophilic derivative coassembled with the PAs in cells that express the Env protein of HIV-1, dialkyl conjugates were more tightly packed than monoalkyl conjugates leading to a less effective disassembly and subsequent association to the cell membrane.

To sum up, our results demonstrated that the length of the PEG, the position of the peptide conjugation, as well as the type of hydrophobic residue determine the antiviral activity of the PA. Although most of them demonstrated moderate antiviral potencies in the cellular model with submicromolar IC_50_ values, *C*-PA_monoalkyl_ showed the highest antiviral activity with IC_50_ in the nanomolar concentration range and an SI index of 250. Moreover, *C*-PA_monoalkyl_ showed the highest affinity for the cell membrane.

These results allow us to correlate the affinity for the cell membrane of PAs with the cellular antiviral activity and agree with the results previously described by Figueira et al.^40^ According to these authors, the antiviral activity of a lipid-conjugated peptide derived from the measles virus fusion protein results from a combination of self-aggregation properties and integration into the target cell membrane. In their study, the lipid-conjugated peptide integration into the membrane of the target cell increased the potency of the FI. In addition, they demonstrated that the stability of the self-assembled nanostructures is an important determinant of the peptide membrane interaction, with highly stable micellar self-assembled structures being inefficient to disassemble upon contact with membranes.^41^ From our results, we speculate that *C*-PA_monoalkyl_ self-assembles into lamellar-like ordering nanostructures that can be disassembled in membranes leading to the exposure of the peptide sequence in the membrane domains where protein-mediated fusion of the virus envelope takes place. Indeed, *C*-PA_monoalkyl_ can aggregate in the form of bilayers when in contact with water; this means that the incorporation of this molecule to a membrane will not deeply alter the curvature. Low curvature aggregates correspond to a low concentration of monomers in solution (as observed in bilayer forming phospholipids), and therefore, the incorporation into the membranes is unlikely to proceed through monomer diffusion but via membrane fusion. This also agrees with the observation of the peptide presence only in the external leaflet of the bilayer in the studies of interaction of the peptides with model membranes.

In our hands, the design of novel amphiphilic peptides made it possible to obtain peptide structures capable of self-assembly in aqueous solution, favoring the solubility of lipopeptides, based on the E1P47 sequence, which are highly hydrophobic and would be highly insoluble under physiological conditions. So far, we have studied the possible administration of entry inhibitor peptides incorporated into drug delivery systems such as liposomes or polymeric nanoparticles.^42−45^ In the present work, we have designed an amphiphilic peptide with anti-HIV-1 activity that is capable of forming self-assembled nanostructures that could allow the administration of the inhibitor peptide without the need of its incorporation into a delivery vehicle.

The order of potency for *N*-PA_monoalkyl_ and *C*-PA_monoalkyl_ against infection with HIV-1_BaL_ was different in mucosal tissue explants than in the cellular screening model of TZM-bl cells. This is not surprising, due to the overexpression of CD4 and CCR5 HIV receptors in the cellular model,^46,47^ which is not a reflection of the *in vivo* levels observed in the mucosal portals of viral entry during sexual intercourse. Furthermore, the activity of the peptides will be affected by accessibility to target cells within the mucosal compartments. The structural complexity of a tissue, the impact of mucosal factors such as secretions and microbiome, as well as transport of peptides through the epithelial barrier cannot be fully mimicked by the TZM-bl cell model. Hence, the tissue explant model is important to validate prescreenings performed in cellular assays such as TZM-bl cells.

In conclusion, the design of peptide amphiphiles-based supramolecular structures can be considered as a suitable strategy to deliver peptides that target viral proteins involved in the fusion process with the cell membrane domains. This work opens new perspectives in the development of novel supramolecular structures based on entry-inhibiting peptides to be applied in the design of broad-spectrum antivirals.

## Experimental Procedures

### Synthesis of Hydrophobic Moieties

#### Synthesis of *N*-Succinyl-octadecylamine

According to the method used by Schmitt et al.,^48^ octadecylamine (0.5 g, 1.855 mmol, 1.0 equiv) and succinic anhydride (0.37 g, 3.710 mmol, 2 equiv) were added to a sealed round-bottom flask under N_2_ atmosphere with balloon. They were dissolved in 40 mL of CHCl_3_ and Et_3_N (2.4 mL, 17.36 mmol); solution was stirred overnight under N_2_ atm. at room temperature. The reaction was followed by TLC in its triethylammonium salt form (CHCl_3_/CH_3_OH 9:1). After the reaction had ended, HCl 1 M was added and a white precipitate appeared. The aqueous medium was lyophilized, and the resulting solid was solved again with CH_2_Cl_2_ (20 mL) and Et_3_N (dropwise until the solid was solved).^49^ Then, it was concentrated in vacuo, and the resulting crystals were crystallized with AcOH overnight. Finally, white crystals were obtained by filtration (0.4792 g, 70% yield).

NMR experiments were acquired at 298 K on a 9.4 T Agilent VNMRS spectrometer operating at 400.13 MHz (1H) equipped with a 5 mm OneNMR probe.

White crystals. *R*_f_ = 0.64 (CH_2_Cl_2_/CH_3_OH 8:1). ^1^H NMR (400 MHz, CDCl_3_) δ 5.68 (s, 1H, NH), 3.28 (td, *J* = 7.1, 5.6 Hz, 2H, NCH_2_), 2.74–2.66 (m, 2H, NCH_2_), 2.58–2.50 (m, 2H, O=CCH_2_), 1.56 (d, *J* = 39.0 Hz, 2H, NCH_2_CH_2_(CH_2_)_15_), 1.29 (d, *J* = 5.1 Hz, 2H), 1.25 (s, 28H), 0.88 (t, *J* = 6.7 Hz, 3H, CH_3_).

#### Synthesis of *N*-Succinyl-dioctadecylamine

Dioctadecylamine (0.5 g, 0.958 mmol, 1.0 equiv) and succinic anhydride (0.19 g, 1.916 mmol, 2 equiv) were added to a sealed round-bottom flask under N_2_ atmosphere with balloon. They were dissolved in 20 mL of CHCl_3_ and Et_3_N (1.2 mL, 8.68 mmol), solution was stirred overnight under N_2_ atm. at room temperature. The reaction was followed by TLC in its triethylammonium salt form (*R*_f_ = 0.66 (CHCl_3_/CH_3_OH 9:1)). After the reaction had ended, a liquid–liquid extraction was performed with HCl 1 M (2 × 30 mL) and H_2_O (3 × 30 mL). Then, it was dried over MgSO_4_ anhydrous, filtrated and concentrated in vacuo. White crystals were obtained (0.5051 g, 85% yield). NMR experiments were acquired at 298 K on a 9.4 T Agilent VNMRS spectrometer operating at 400.13 MHz (1H) equipped with a 5 mm OneNMR probe.

White crystals. *R*_f_ = 0.8 (CHCl_3_/CH_3_OH 9:1). ^1^H NMR (400 MHz, CDCl_3_) δ 3.27 (dt, *J* = 33.6, 7.7 Hz, 4H, (CH_2_)_16_CH_2_N), 2.68 (s, 4H, NHCOCH_2_CH_2_COO^–^), 1.64–1.47 (m, 4H, (CH_2_)_15_CH_2_CH_2_), 1.25 (s, 60H, CH_3_(CH_2_)_17_), 0.88 (t, *J* = 6.8 Hz, 6H, CH_3_).

#### Synthesis of Cholest-5-en-3-yl bromoacetate

The cholesterol derivative was prepared following the procedure described in ref (12). Briefly, cholesterol (0.100 g, 0.259 mmol, 1.0 equiv), bromoacetic acid (0.039 g, 0.284 mmol, 1.1 equiv), and 4-dimethylaminopyridine (DMAP) (0.0035 g, 0.029 mmol, 0.125 equiv) were solved in anhydrous dichloromethane (CH_2_Cl_2_) under argon atmosphere. Then, *N,N*-diisopropylcarbodiimide (DIPCDI) (0.0 mL, 0.310 mmol, 1.2 equiv) was added to the solution that was left stirring at room temperature for 48 h under N_2_. The reaction was analyzed by TLC using 30% CH_2_Cl_2_ in hexane as eluent. The organic phase was washed with pure water to remove salts and subsequently dried with MgSO_4_ anhydrous. The crude was purified on a silica gel column eluting with different fractions of CH_2_Cl_2_ in hexane. After testing the presence of the compound by TLC, the fractions ranging between 20% and 28% of CH_2_Cl_2_ in hexane were collected, and the solvent was removed under vacuum. White crystals were obtained (0.105 g, 80% yield).

NMR experiments were acquired at 298 K on a 11.7 T Bruker AVANCE IIIHD spectrometer operating at 500.13 MHz (1H) equipped with a 5 mm cryogenically cooled triple-resonance probehead (TCI) (Figure S10, Supporting Information).

### Synthesis of N-Peptide Amphiphiles

The synthesis of the E1P47 peptide sequence (WILEYLWKVPFDFWRGVI) was carried out on solid phase as described in ref (17). The peptide was manually synthesized on a NovaSyn TGR resin (1.5 g, 0.22 mmol.g^–1^) as C-terminal carboxamides and following the Fmoc/tBu orthogonal protection strategy. The coupling reaction was performed using 3-fold molar excesses of activated Fmoc-amino acids (0.99 mmol, 3 equiv) throughout the synthesis. The protected amino acid derivatives were activated by treatment with HATU (0.376 g, 0.99 mmol, 3 equiv) and DIEA (0.345 mL, 1.98 mmol, 6 equiv). The deprotection of Fmoc was accomplished twice with 20% (v/v) piperidine in DMF for 10 min. All coupling and deprotection steps were checked by the Kaiser test, based on the reaction of ninhydrin with primary amines, or chloranil test that allows reliable detection of secondary amino groups. Upon finishing the synthesis of the peptide sequence, the functionality of the peptidyl-resin was 0.129 mmol/g. Subsequently, *N*-peptide amphiphiles represented in Figure 1 were synthesized from this peptidyl-resin. Detailed synthetic procedures as well as chemical characterization of purified products are reported in Supporting Information.

### Synthesis of C-Peptide Amphiphiles

The synthesis of *C*-PA_monoalkyl_, *C*-PA_dialkyl_, and *C*-PA_chol_ was carried out on a NovaSyn TGR R resin (0.510 g, 0.19 mmol.g^–1^) as C-terminal carboxamides and following the Fmoc/tBu strategy. Regarding the derivatization of the E1P47 on C-terminus, the orthogonal Lys derivative protected at Nε- with the 4-methyltrityl group (Mtt), Fmoc-Lys(Mtt)-OH, was introduced at the C-terminal position of the peptide sequence. The coupling reactions were performed using 3-fold molar excesses of activated Fmoc-amino acids throughout the synthesis (0.29 mmol, 3 equiv). The protected amino acid derivatives were activated by treatment with HATU (0.29 mmol, 3 equiv) and DIEA (0.58 mmol, 6 equiv). The deprotection of Fmoc was accomplished twice with 20% (v/v) piperidine in DMF for 10 min twice. Upon finishing the synthesis of the peptide sequence, the functionality of the peptidyl-resin was 0.113 mmol.g^–1^.

Three fractions of Fmoc-deprotected E1P47 peptidyl-resin (0.25 g, 0.028 mmol, 1.0 equiv) were weighed in 20 mL reservoirs and solvated overnight with DMF. Once the resins were swelled, the solvent was removed, a solution of CH_2_Cl_2_ 95% (v/v), TIS 4% (v/v), and TFA 1% (v/v) was prepared for the selective deprotection of Mtt group. The peptidyl-resins were treated 5 times for 15 min with the solution and the last time for 2 h. Then, they were rinsed with DMF × 5 and the Kaiser test was performed for each peptidyl-resin. Afterward, a mixture of Fmoc-NH-PEG_27_-COOH (0.0865 g, 0.056 mmol, 2 equiv), PyBOP (0.029 g, 0.056 mmol, 2 equiv), HOBt (0.0076 g, 0.056 mmol, 2 equiv), and DIEA (0.019 mL, 0.109 mmol, 4 equiv) was solved in the minimum amount of DMF and was added to each reactor. The reactions took place overnight at room temperature. The subsequent derivatizations with the hydrophobic moieties as well as the chemical characterization of the final products are detailed in the Supporting Information.

### Synthesis of K-peptide amphiphiles

*K*-PA_monoalkyl_, *K*-PA_dialkyl_, and *K*-PA_chol_ derivatives were manually synthesized on a NovaSyn TGR resin (1.0 g, 0.22 mmol.g^–1^). The coupling reactions were performed using 3-fold molar excesses of activated Fmoc-amino acids throughout the synthesis (0.66 mmol, 3 equiv). The protected amino acid derivatives were activated by treatment with HATU (0.66 mmol, 3 equiv) and DIEA (1.32 mmol, 6 equiv). The Lys residue at position 8 of the peptide sequence was introduced using the N-α-Fmoc-N-ε-4-methyltrityl-l-lysine (Fmoc-Lys(Mtt)-OH) derivative. Upon finishing the synthesis of the peptide sequence, the functionality of the peptidyl-resin was 0.129 mmol.g^–1^.

Three fractions of Fmoc-deprotected E1P47 peptidyl-resin (0.2 g, 0.026 mmol, 1.0 equiv) were weighed in three 20 mL reservoirs and solvated overnight with DMF. The selective deprotection of Mtt group was performed as described for the synthesis in the C-derivatized E1P47. Afterward, a mixture of Fmoc-NH-PEG_27_-COOH (0.080 g, 0.052 mmol, 2 equiv), PyBOP (0.027 g, 0.052 mmol, 2 equiv), HOBt (0.007 g, 0.052 mmol, 2 equiv), and DIEA (0.018 mL, 0.104 mmol, 4 equiv) was solved in the minimum amount of DMF (∼5 mL). Then, the solution was added to each reactor and the reaction took place overnight at room temperature. The subsequent derivatizations with the hydrophobic moieties (Figure 4) as well as the chemical characterization of the final products are detailed in the Supporting Information.

### Fluorescence Assays

Fluorescence emission spectra were recorded in a PTI Fluorescence Master Systems spectrofluorometer (Photon Technology International, Birmingham, AL, USA) equipped with a temperature controller in 1.0 cm path length quartz cuvette (Hellma Analytics, Müllheim, Germany). Intrinsic fluorescence emission of the tryptophan residues was performed at a peptide concentration of 5.0 × 10^–6^ M in HEPES buffer (0.01 M, pH 7.4) at 25 °C using an excitation wavelength of 280 nm.

In addition, emission fluorescence spectra were recorded for each PA upon titration with increasing concentrations of POPC liposomes to carry out membrane partition studies with the peptide conjugates. Unilamellar vesicles (LUV) of POPC were prepared as previously described.^50^ Changes in the intrinsic fluorescence spectra of tryptophan residues were monitored after incubation of 5.0 × 10^–6^ M in HEPES buffer (0.01 M, pH 7.4) with increasing concentrations of POPC liposomes (concentration ranging from 12.5 × 10^–6^ M to 2.0 × 10^–4^ M). The suspensions were continuously stirred and were left to equilibrate for 10 min before recording the emission spectra at an excitation wavelength of 280 nm. Fluorescence intensities were corrected by subtraction of the vesicle blank. A lipid titration of N-acetyltryptophanamide (NATA) was carried out at the same time to account for the dilution effect as well as for any variations due to lamp power fluctuations. The apparent mole fraction partition coefficients were determined as previously described.^50^

### Surface Tension Measurements

Surface tension was measured using a homemade pendant droplet instrument as described by Pi-Boleda et al.^51^ Briefly, aqueous (0.5% DMSO) solutions of the peptides were prepared at a concentration of 20 μM. Those solutions were visually inspected to discard the presence of precipitates and were further diluted with 3% DMSO. Those aliquots were set to hang at the end of straight cut Teflon tubes with internal/external diameters of 0.8:1.6 mm. The image was recorded, and the profile was extracted using a maximum gradient algorithm. The profile was fitted to the Young–Laplace equation using a Golden Section search strategy. The system was kept at saturation humidity and thermostated at 25 ± 0.2 °C. Pictures were taken until the surface tension did not show systematic drift (typically 3–4 h).

### Small Angle X-ray Scattering (SAXS)

Two different instruments were used for the measurements. An in-house instrument from Hecus at the SAXS-WAXS service at IQAC and the NCD-Sweet beamline at Synchrotron ALBA.

The Hecus instrument details are given, for instance, in Haba et al.^35^ We have used a linear position detector. In these conditions, the spectra are mainly smeared by the convolution of the scattered beam with the detector width. This has been taken into account for model fitting. SAXS range was calibrated with silver behenate.

NCD-Sweet was set up using a sample to detector distance of 2945 mm and using 0.099987 nm radiation wavelength. SAXS was calibrated using silver behenate. The DOPC and DOPC doped samples were introduced in a flowthrough 1-mm-diameter capillary. 3% DMSO in water was used as subtracting background.

We used a symmetric Gaussian based model for describing the bilayer electronic profile as detailed in Pabst et al.^36^ with small modifications as found in Haba et al.^35^ Further, to give account of asymmetric bilayers, the asymmetric profile was numerically Fourier transformed while keeping the same description for the interaction of bilayers as before. This asymmetry was introduced in two ways: in a first approach, starting from the parameters of the symmetric parameters obtained with nondoped bilayers, a dissymmetry factor for width, height, and position of one of the leaflets was allowed to vary. In a second approach, to the symmetric bilayer obtained from the fit of the nondoped bilayer, a three slabs profile was added; this corresponds to the addition of 7 new parameters compared to the 3 additional parameters of the Gaussian model. Both models satisfactorily reduced the discrepancy with the experimental data; however, only the second produced results compatible with the known chemical composition of the additives.

Humected PAs samples were fitted to three slab symmetric models built as the methylene slab of the Gaussian model^35^ or to globular models with step functions with interaction via Hard Spheres model.

### Cellular and Mucosal Tissue Assays

#### Toxicity Assessment

The metabolic activity of the cell line, related to the cytotoxic effect of the compounds to be tested, is measured after 24 h using the MTT assay. This assay is based on the metabolic reduction of 3-(4,5-dimethylthiazol-2-yl)-2,5-diphenyl-2*H*-tetrazolium bromide (MTT), carried out by the mitochondrial enzyme succinate dehydrogenase, in a colored compound of blue color (formazan) that can be monitored by absorbance spectroscopy at 560 nm, allowing determination of the mitochondrial functionality of the treated cells.

The HeLa-env cell line is seeded in 96-well plates with an initial density of 15,000 cells per well in 100 μL of culture medium (Dulbecco’s modified Eagle’s medium, DMEM, with 10% FBS added) and incubated for 24 h at 37 °C and 5% CO_2_ to allow their adhesion. The cell monolayers are washed with PBS, and then 100 μL of PAs formulations (peptide amphiphiles coassembled with 5% FAM) are added in a concentration range from 40 μM to 2.3 μM. The plates are kept in incubation for further 24 h.

At the end of the incubation time, the cells are incubated for 3 h with 1 mg/mL of MTT, dissolved in culture medium without FBS, to allow the formation of formazan crystals. Subsequently, 100 μL of DMSO are added to wells, stirring the plates for 30 min at room temperature to facilitate the complete dissolution of the crystals. Finally, the absorbance at 560 nm is measured in a microplate reader.

The results are expressed as relative percentage between absorbance of PA formulations against absorbance of its DMSO controls and CC_50_ and CC_25_ values are taken from dose–response curves.

#### Cellular Uptake of PAs by Flow Cytometry

Cellular internalization of FAM-labeled PAs was quantified through flow cytometry by using a Guava EasyCyteTM system (Millipore Corporation, Hayward, USA).

Briefly, HeLa-env cells were seeded in 12-well plates at 10^5^ cells per well and incubated in cell culture conditions for 24 h in DMEM medium supplemented with 10% FBS. Afterward, cells were rinsed with phosphate buffered saline (PBS) and replenished with 500 μL DMEM FBS-free media containing 5% FAM-labeled PAs at a concentration of 6 μM. In addition, 5% FAM-labeled E1P47 peptide was performed. The cells were incubated for 4 h, and then the whole cell medium was removed. Subsequently, cells were washed thrice with PBS, harvested by trypsinization, centrifuged, and resuspended in 200 μL of fresh PBS.

In order to distinguish between FAM-labeled PAs internalized by cells from those adsorbed to the exterior of cell membrane, a trypan blue solution (0,2%, w/w) was added to the cellular suspension and incubated for 5 min at room temperature, Then, a new step of washed with PBS followed by centrifugation and resuspension with 200 μL of PBS is required.

Cell viability was checked with propidium iodide (2 μg/mL) added after the final resuspension steps, and the cells were then analyzed by flow cytometry in quadruplicate (two replicates were incubated with trypan blue and the other two in absence). Data acquisition was performed in InCite software where 10,000 events were recorded in the gated regions of interest assigned to HeLa-env cells.

#### Cellular Distribution of PAs Using Fluorescence Microscopy

Cellular distribution of FAM-labeled PAs was monitored through optical fluorescence microscopy using an EVOS M7000 Imaging System (Thermo Scientific, USA).

Briefly, HeLa-env cells were seeded in 12-well plates at 10^5^ cells per well and incubated in cell culture conditions for 24 h in DMEM medium supplemented with 10% FBS. Afterward, cells were rinsed with phosphate buffered saline (PBS) and replenished with 500 μL DMEM FBS-free media containing 5% FAM-labeled *C*-PA_monoalkyl_ at a concentration of 6 μM. In addition, 5% FAM-labeled E1P47 peptide was performed. The cells were incubated for 4 h, and then the whole cell medium was removed. Subsequently, cells were washed thrice with 500 μL PBS, and fresh PBS was added before capturing images in the microscope.

Plates were placed in the microscope, kept at room temperature, and images were captured using a 40× objective and GFP channel fluorescence (470/22 nm excitation filter and 510/42 nm emission filter) suitable for 5(6)-carboxyfluorescein (FAM) dye.

#### Anti-HIV-1 Activity of PAs

HIV-1_BaL_^52^ was provided by the NIH AIDS Research & Reference Reagent Program (http://www.aidsreagent.org/). Viral stock was prepared by passage through activated PBMCs^53^ for 11 days.

Luciferase-reporter HeLa cells stably transfected to express CD4 and CCR5; TZM-bl cells^54−56^ were grown in Dulbecco’s Minimal Essential Medium (DMEM) (Sigma-Aldrich, Inc., St. Louis, MO) containing 10% fetal calf serum (FCS), 2 mM l-glutamine, and antibiotics (100 U of penicillin/mL, 100 μg of streptomycin/mL) at 37 °C in an atmosphere containing 5% CO_2_.

Human tissue explants: Surgically resected specimens of colorectal tissues were collected from HIV negative patients at St. Mary’s Hospital, Imperial College Healthcare NHS Trust, London, UK. All tissues were collected after receiving signed informed consent from all patients through the Imperial College Healthcare Tissue Bank approved by Research Ethics Committee Wales (IRAS 17/WA/0161). On arrival in the laboratory, resected tissue was cut into 2–3 mm^3^ explants comprising both epithelial and muscularis mucosae as described previously.^57^ Tissue explants were maintained with DMEM containing 10% fetal calf serum, 2 mM l-glutamine, and antibiotics (100 U of penicillin/mL, 100 μg of streptomycin/mL, 80 μg of gentamicin/mL) at 37 °C in an atmosphere containing 5% CO_2_.

All inhibition assays in cellular and tissue explants models were performed using a standardized amount of virus culture supernatant normalized for infectivity. TZM-bl cells were incubated with serial dilutions of peptides (from 10 μM to 0.00061 μM) for 1 h at 37 °C, prior to challenge with HIV-1_BaL_. Luciferase expression (r.l.u. values) was determined after 48 h. Alternatively, tissue explants were incubated with serial dilutions of peptide (from 100 μM to 0.1 μM) for 1 h before virus (10^4^ TCID_50_/mL) was added for 2 h. Explants were then washed four times with PBS to remove unbound virus and drug. Colorectal explants were then transferred onto gelfoam rafts (Welbeck Pharmaceuticals, UK) and cultured for 15 days as described previously^57^ in the presence or absence of drug. Explants were cultured for up to 15 days in the absence of peptide. Approximately 50% of the supernatants were harvested every 2–3 days and explants were re-fed with fresh media. The extent of viral replication in tissue explants was determined by measuring the p24 antigen concentration in supernatants (INNOTEST HIV antigen mAb; Fujirebio Europe, Belgium). The extent of inhibition by each peptide was calculated. The percentage of inhibition was normalized relative to the r.l.u, for TZM-bl cells, or p24, for tissue explants, values obtained for cells or explants cultured in the absence of virus (100% inhibition) and for cells or explants infected with virus in the absence of peptide (0% inhibition). Data are the mean (±SEM) of triplicates. When a dose–response curve was obtained, IC_50_ and, if possible, IC_95_ values were calculated from sigmoid curve fits (GraphPad Prism). For all curves, *R*^2^ is >0.7.

## Supporting Information Available

Experimental procedures, primary structure of PAs, HIV-1 inhibitory activity, fluorescence emission spectra, surface tension, SAXS, partitioning isotherms, cytotoxic concentration values, and fitting parameters (PDF)

## Abbreviations

FI: fusion inhibitor
FDA: Food and Drug Administration
HIV-1: human immunodeficiency virus type 1
PA: peptide amphiphile
PEG: polyethylene glycol
Fmoc: 9-fluorenylmethoxycarbonyl
tBut: *tert*-butyl
ES-MS: electrospray mass spectrometry
IC_50_: half-maximal inhibition concentration
IC_95_: concentration of 95% inhibition
DMSO: dimethyl sulfoxide
CMC: critical micellar concentration
FAM: 5(6)-carboxyfluorescein
POPC: 1-palmitoyl-2-oleoyl-*sn*-glycero-3-phosphocholine
SAXS: small angle X-ray scattering
SI: selective index
CC: cytotoxic concentration
Env: envelope
TLC: thin layer chromatography
PBS: phosphate buffered saline

## References

[ref1] Pattnaik G. P.; Chakraborty H. (2020) Entry Inhibitors: Efficient Means to Block Viral Infection. J. Membr. Biol. 253 (5), 425–444. 10.1007/s00232-020-00136-z. 32862236 PMC7456447

[ref2] Malik T.; Chauhan G.; Rath G.; Murthy R. S. R.; Goyal A. K. (2017) Fusion and binding inhibition” key target for HIV-1 treatment and pre-exposure prophylaxis: targets, drug delivery and nanotechnology approaches. Drug Delivery 24 (1), 608–621. 10.1080/10717544.2016.1228717. 28240046 PMC8241151

[ref3] Verma J.; Subbarao N.; Rajala M. S. (2020) Envelope proteins as antiviral drug target. J. Drug Target. 28 (10), 1046–1052. 10.1080/1061186X.2020.1792916. 32643453

[ref4] Al-Azzam S.; Ding Y.; Liu J.; Pandya P.; Ting J. P.; Afshar S. (2020) Peptides to combat viral infectious diseases. Peptides 134, 170402 10.1016/j.peptides.2020.170402. 32889022 PMC7462603

[ref5] Acar H.; Srivastava S.; Chung E. J.; Schnorenberg M. R.; Barrett J. C.; LaBelle J. L.; Tirrell M. (2017) Self-assembling peptide-based building blocks in medical application. Adv. Drug Delivery Rev. 110–111, 65–79. 10.1016/j.addr.2016.08.006. PMC5922461 27535485

[ref6] Su X.; Wang Q.; Wen Y.; Jiang S.; Lu L. (2020) Protein and Peptide-Based Virus Inactivators: Inactivating Viruses Before Their Entry Into Cells. Front. Microbiol. 11, 1063 10.3389/fmicb.2020.01063. 32523582 PMC7261908

[ref7] Pu J.; Wang Q.; Xu W.; Lu L.; Jiang S. (2019) Development of Protein- and Peptide-Based HIV Entry Inhibitors Targeting gp120 or gp41. Viruses 11 (8), 705 10.3390/v11080705. PMC6722851 31374953

[ref8] Gómara M. J.; Haro I. (2014) Updating the Use of Synthetic Peptides as Inhibitors of HIV-1 Entry. Curr. Med. Chem. 21, 1188–1200. 10.2174/15672050113109990204. 23931277

[ref9] Burton A. (2003) Enfuvirtide approved for defusing HIV. Lancet Infect. Dis. 3 (5), 260 10.1016/S1473-3099(03)00620-0. 12726955

[ref10] Tang X.; Jin H.; Chen Y.; Li L.; Zhu Y.; Chong H.; He Y. (2019) A membrane-anchored short-peptide fusion inhibitor fully protects target cells from infections of human immunodeficiency virus type 1 (HIV-1), HIV-2, and simian immunodeficiency virus. J. Virol. 93, e01177–19. 10.1128/JVI.01177-19. PMC6819927 31462566

[ref11] Wexler-Cohen Y.; Shai Y. (2009) Membrane-Anchored HIV-1 N-Heptad Repeat Peptides Are Highly Potent Cell Fusion Inhibitors via an Altered Mode of Action. PLoS Pathog. 5 (7), e1000509 10.1371/journal.ppat.1000509. 19593361 PMC2699469

[ref12] Ingallinella P.; Bianchi E.; Ladwa N. A.; Wang Y.-J.; Hrin R.; Veneziano M.; Bonelli F.; Ketas T. J.; Moore J. P.; Miller M. D.; et al. (2009) Addition of a cholesterol group to an HIV-1 peptide fusion inhibitor dramatically increases its antiviral potency Proc. Proc. Natl. Acad. Sci. U. S. A. 106 (14), 5801–5806. 10.1073/pnas.0901007106. 19297617 PMC2667053

[ref13] Harman S.; Herrera C.; Armanasco N.; Nuttall J.; Shattock R. J. (2012) Preclinical evaluation of the HIV-1 fusion inhibitor L’644 as a potential candidate microbicide. Antimicrob. Agents Chemother. 56 (5), 2347–2356. 10.1128/AAC.06108-11. 22330930 PMC3346626

[ref14] Ding X.; Zhang X.; Chong H.; Zhu Y.; Wei H.; Wu X.; He J.; Wang X.; He Y. (2017) Enfuvirtide T20-based lipopeptide is a potent HIV-1 cell fusion inhibitor: implications for viral entry and inhibition. J. Virol. 91, e00831–17. 10.1128/JVI.00831-17. 28659478 PMC5571253

[ref15] Zhu Y.; Zhang X.; Ding X.; Chong H.; Cui S.; He J.; Wang X.; He Y. (2018) Exceptional potency and structural basis of a T1249-derived lipopeptide fusion inhibitor against HIV-1, HIV-2, and simian immunodeficiency virus. J. Biol. Chem. 293 (14), 5323–5334. 10.1074/jbc.RA118.001729. 29425101 PMC5892594

[ref16] Chong H.; Xue J.; Zhu Y.; Cong Z.; Chen T.; Wei Q.; Qin C.; He Y. (2019) Monotherapy with a low-dose lipopeptide HIV fusion inhibitor maintains long term viral suppression in rhesus macaques. PLoS Pathog. 15 (2), e1007552 10.1371/journal.ppat.1007552. 30716118 PMC6375636

[ref17] Gómara M. J.; Sánchez-Merino V.; Paús A.; Merino-Mansilla A.; Gatell J. M.; Yuste E.; Haro I. (2016) Definition of an 18-mer Synthetic Peptide Derived from the GB virus C E1 Protein as a New HIV-1 Entry Inhibitor. Biochim. Biophys. Acta, Gen. Subj. 1860 (6), 1139–1148. 10.1016/j.bbagen.2016.02.008. 26905802

[ref18] Pérez Y.; Gómara M. J.; Yuste E.; Gómez-Gutierrez P.; Pérez J. J.; Haro I. (2017) Structural Study of a New HIV-1 Entry Inhibitor and Interaction with the HIV-1 Fusion Peptide in Dodecylphosphocholine Micelles. Chem. - Eur. J. 23, 11703–11713. 10.1002/chem.201702531. 28677862

[ref19] Raymond D. M.; Nilsson B. L. (2018) Multicomponent peptide assemblies. Chem. Soc. Rev. 47 (10), 3659–3720. 10.1039/C8CS00115D. 29697126 PMC6538069

[ref20] Edwards-Gayle C. J. C.; Hamley I. W. (2017) Self-assembly of bioactive peptides, peptide conjugates, and peptide mimetic materials. Org. Biomol. Chem. 15 (28), 5867–5876. 10.1039/C7OB01092C. 28661532

[ref21] Guyon L.; Lepeltier E.; Passirani C. (2018) Self-assembly of peptide-based nanostructures: Synthesis and biological activity. Nano Res. 11 (5), 2315–2335. 10.1007/s12274-017-1892-9.

[ref22] Hamley I. W. (2014) PEP-peptide conjugates. Biomacromolecules 15, 1543–1559. 10.1021/bm500246w. 24720400

[ref23] Pike L. J. (2003) Lipid rafts: bringing order to chaos. J. Lipid Res. 44 (4), 655–667. 10.1194/jlr.R200021-JLR200. 12562849

[ref24] Gomara M. J.; Galatola R.; Gutiérrez A.; Gimeno M. C.; Gatell J. M.; Sánchez-Merino V.; Yuste E.; Haro I. (2013) HIV-1 inhibiting capacity of novel forms of presentation of GB virus C peptide domains is enhanced by coordination to gold compounds. Curr. Med. Chem. 21, 238–250. 10.2174/09298673113206660276. 24083612

[ref25] Pfefferkorn C. M.; McGlinchey R. P.; Lee J. C. (2010) Effects of pH on aggregation kinetics of the repeat domain of a functional amyloid, Pmel17. Proc. Natl. Acad. Sci. U. S. A. 107 (50), 21447–21452. 10.1073/pnas.1006424107. 21106765 PMC3003087

[ref26] Chaari A.; Fahy C.; Chevillot-Biraud A.; Rholam M. (2015) Insights into Kinetics of Agitation-Induced Aggregation of Hen Lysozyme under Heat and Acidic Conditions from Various Spectroscopic Methods. PLoS One 10 (11), e0142095 10.1371/journal.pone.0142095. 26571264 PMC4646502

[ref27] Lin D.; Ren R.; Tan Q.; Wu Q.; Li F.; Li L.; Liu S.; He J. (2016) A facile and dynamic assay for the detection of peptide aggregation. Anal. Bioanal. Chem. 408, 1609–1614. 10.1007/s00216-015-9271-4. 26738494

[ref28] Toprakcioglu Z.; Challa P.; Xu C.; Knowles T. P. J. (2019) Label-Free Analysis of Protein Aggregation and Phase Behavior. ACS Nano 13 (12), 13940–13948. 10.1021/acsnano.9b05552. 31738513

[ref29] Ladokhin A. S.; Jayasinghe S.; White S. H. (2000) How to Measure and Analyze Tryptophan Fluorescence in Membranes Properly, and Why Bother? Anal. Anal. Biochem. 285 (2), 235–245. 10.1006/abio.2000.4773. 11017708

[ref30] Li P. X.; Thomas R. K.; Penfold J. (2014) Limitations in the Use of Surface Tension and the Gibbs Equation to Determine Surface Excesses of Cationic Surfactants. Langmuir 30, 6739–6747. 10.1021/la501287v. 24853780

[ref31] Schick M. J. (1966) Micelle formation in mixtures of nonionic and cationic detergents. J. Am. Oil Chem. Soc. 43, 681–682. 10.1007/BF02682570.

[ref32] Barry B. W.; Morrison J. C.; Russell G. F. (1970) Prediction of the critical micelle concentration of mixtures of alkyltrimethylammonium salts. J. Colloid Interface Sci. 33, 554–561. 10.1016/0021-9797(70)90007-X.

[ref33] Rodríguez J. L.; Sierra M. B.; Messina P. V.; Morini M. A.; Schulz P. C.; del Burgo P.; Junquera E.; Rodríguez A.; Aicart E. (2007) Surface and bulk properties of aqueous decyltrimethylammonium bromide–hexadecyltrimethylammonium bromide mixed system. J. Colloid Interface Sci. 314, 699–706. 10.1016/j.jcis.2007.06.010. 17658541

[ref34] Pucci C.; Pérez L.; La Mesa C.; Pons R. (2014) Characterization and stability of catanionic vesicles formed by pseudo-tetraalkyl surfactant mixtures. Soft Matter 10, 9657–9667. 10.1039/C4SM01575D. 25356774

[ref35] Haba E.; Pinazo A.; Pons R.; Pérez L.; Manresa A. (2014) Complex rhamnolipid mixture characterization and its influence on DPPC bilayer organization. Biochim. Biophys. Acta, Biomembr. 1838, 776–783. 10.1016/j.bbamem.2013.11.004. 24239913

[ref36] Pabst G.; Rappolt M.; Amenitsch H.; Laggner P. (2000) Structural information from multilamellar liposomes at full hydration: full q-range fitting with high quality X-ray data. Phys. Rev. E: Stat. Phys., Plasmas, Fluids, Relat. Interdiscip. Top. 62, 4000–4009. 10.1103/PhysRevE.62.4000. 11088921

[ref37] Frampton M. B.; Marquardt D.; Letofsky-Papst I.; Pabst G.; Zelisko P. M. (2017) Analysis of Trisiloxane Phosphocholine Bilayers. Langmuir 33, 4948–4953. 10.1021/acs.langmuir.6b04162. 28471667 PMC5462096

[ref38] Castangia I.; Manca M. L.; Caddeo C.; Maxia A.; Murgia S.; Pons R.; Demurtas D.; Pando D.; Falconieri D.; Peris J. E.; et al. (2015) Faceted phospholipid vesicles tailored for the delivery of *Santolina insularis* essential oil to the skin. Colloids Surf., B 132, 185–193. 10.1016/j.colsurfb.2015.05.025. 26057243

[ref39] Kucerka N.; Nieh M. P.; Katsaras J. (2009) Asymmetric distribution of cholesterol in unilamellar vesicles of monounsaturated phospholipids. Langmuir 25, 13522–13527. 10.1021/la9020299. 19678653

[ref40] Figueira T. N.; Palermo L. M.; Veiga A. S.; Huey D.; Alabi C. A.; Santos N. C.; Welsch J. C.; Mathieu C.; Horvat B.; Niewiesk S.; et al. (2017) In Vivo Efficacy of Measles Virus Fusion Protein-Derived Peptides Is Modulated by the Properties of Self-Assembly and Membrane Residence. J. Virol. 91 (1), e01554–16. 10.1128/JVI.01554-16. 27733647 PMC5165226

[ref41] Figueira T. N.; Mendonça D. A.; Gaspar D.; Melo M. N.; Moscona A.; Porotto M.; Castanho M. A. R. B.; Veiga A. S. (2018) Structure–Stability–Function Mechanistic Links in the Anti-Measles Virus Action of Tocopherol-Derivatized Peptide Nanoparticles. ACS Nano 12, 9855–9865. 10.1021/acsnano.8b01422. 30230818 PMC6399014

[ref42] Gómara M. J.; Pérez-Pomeda I.; Gatell J. M.; Sánchez-Merino V.; Yuste E.; Haro I. (2017) Lipid raft-like liposomes used for targeted delivery of a chimeric entry-inhibitor peptide with anti-HIV-1 activity. Nanomedicine 13 (2), 601–609. 10.1016/j.nano.2016.08.023. 27565689

[ref43] Ariza-Sáenz M.; Espina M.; Bolaños N.; Calpena A. C.; Gómara M. J.; Haro I.; García M. L. (2017) Penetration of polymeric nanoparticles loaded with an HIV-1 inhibitor peptide derived from GB virus C in a vaginal mucosa model. Eur. J. Pharm. Biopharm. 120, 98–106. 10.1016/j.ejpb.2017.08.008. 28842284

[ref44] Ariza-Sáenz M.; Espina M.; Calpena A. C.; Gómara M. J.; Pérez-Pomeda I.; Haro I.; García M. L. (2018) Design, Characterization, and Biopharmaceutical Behavior of Nanoparticles Loaded with an HIV-1 Fusion Inhibitor Peptide. Mol. Pharmaceutics 15, 5005–5018. 10.1021/acs.molpharmaceut.8b00609. 30226777

[ref45] Sanchez-Lopez E.; Paus A.; Perez-Pomeda I.; Calpena A.; Haro I.; Gomara M. J. (2020) Lipid Vesicles Loaded with an HIV-1 Fusion Inhibitor Peptide as a Potential Microbicide. Pharmaceutics 12, 502 10.3390/pharmaceutics12060502. PMC7355883 32486415

[ref46] Polonis V. R.; Brown B. K.; Rosa Borges A.; Zolla-Pazner S.; Dimitrov D. S.; Zhang M. Y.; Barnett S. W.; Ruprecht R. M.; Scarlatti G. (2008) Recent advances in the characterization of HIV-1 neutralization assays for standardized evaluation of the antibody response to infection and vaccination. Virology 375, 315–320. 10.1016/j.virol.2008.02.007. 18367229

[ref47] Platt E. J.; Wehrly K.; Kuhmann S. E.; Chesebro B.; Kabat D. (1998) Effects of CCR5 and CD4 cell surface concentrations on infections by macrophagetropic isolates of human immunodeficiency virus type 1. J. Virol. 72, 2855–2864. 10.1128/JVI.72.4.2855-2864.1998. 9525605 PMC109730

[ref48] Schmitt L.; Dietrich C.; Tampé R. (1994) Synthesis and Characterization of Chelator-Lipids for Reversible Immobilization of Engineered Proteins at Self-Assembled Lipid Interfaces. J. Am. Chem. Soc. 116, 8485–8491. 10.1021/ja00098a008.

[ref49] Korneev S.; Rosemeyer H. (2013) Synthesis of functionalized lipids, and their use for a tunable hydrophobization of nucleosides and nucleic acids. Helv. Chim. Acta 96, 201–216. 10.1002/hlca.201100410.

[ref50] Gomara M. J.; Perez Y.; Martinez J. P.; Barnadas-Rodriguez R.; Schultz A.; von Briesen H.; Peralvarez-Marin A.; Meyerhans A.; Haro I. (2019) Peptide Assembly on the Membrane Determines the HIV-1 Inhibitory Activity of Dual-Targeting Fusion Inhibitor Peptides. Sci. Rep. 9, 3257 10.1038/s41598-019-40125-4. 30824796 PMC6397244

[ref51] Pi-Boleda B.; Sorrenti A.; Sans M.; Illa O.; Pons R.; Branchadell V.; Ortuño R. M. (2018) Cyclobutane scaffold in bolaamphiphiles: effect of diastereoisomerism and regiochemistry on their surface activity aggregate structure. Langmuir 34, 11424–11432. 10.1021/acs.langmuir.8b01462. 30173523

[ref52] Gartner S.; Markovits P.; Markovitz D. M.; Kaplan M. H.; Gallo R. C.; Popovic M. (1986) The role of mononuclear phagocytes in HTLV-III/LAV infection. Science 233, 215–219. 10.1126/science.3014648. 3014648

[ref53] Gordon C. J.; Muesing M. A.; Proudfoot A. E.; Power C. A.; Moore J. P.; Trkola A. (1999) Enhancement of Human Immunodeficiency Virus Type 1 Infection by the CC-Chemokine RANTES Is Independent of the Mechanism of Virus-Cell Fusion. J. Virol. 73, 684–694. 10.1128/JVI.73.1.684-694.1999. 9847374 PMC103875

[ref54] Derdeyn C. A.; Decker J. M.; Sfakianos J. N.; Wu X.; O’Brien W. A.; Ratner L.; Kappes J. C.; Shaw G. M.; Hunter E. (2000) Sensitivity of human immunodeficiency virus type 1 to the fusion inhibitor T-20 is modulated by coreceptor specificity defined by the V3 loop of gp120. J. Virol. 74, 8358–8367. 10.1128/JVI.74.18.8358-8367.2000. 10954535 PMC116346

[ref55] Platt E. J.; Wehrly K.; Kuhmann S. E.; Chesebro B.; Kabat D. (1998) Effects of CCR5 and CD4 cell surface concentrations on infections by macrophagetropic isolates of human immunodeficiency virus type 1. J. Virol. 72, 2855–2864. 10.1128/JVI.72.4.2855-2864.1998. 9525605 PMC109730

[ref56] Wei X.; Decker J. M.; Liu H.; Zhang Z.; Arani R. B.; Kilby J. M.; Saag M. S.; Wu X.; Shaw G. W.; Kappes J. C. (2002) Emergence of resistant human immunodeficiency virus type 1 in patients receiving fusion inhibitor (T-20) monotherapy. Antimicrob. Agents Chemother. 46, 1896–1905. 10.1128/AAC.46.6.1896-1905.2002. 12019106 PMC127242

[ref57] Herrera C.; Cranage M.; McGowan I.; Anton P.; Shattock R. J. (2009) Reverse transcriptase inhibitors as potential colorectal microbicides. Antimicrob. Agents Chemother. 53 (5), 1797–1807. 10.1128/AAC.01096-08. 19258271 PMC2681527

