# Supplementary material for: Peptide Amphiphilic-Based Supramolecular Structures with Anti-HIV-1 Activity
Source: Bioconjug Chem. 2021 Jul 13;32(9):1999–2013. doi: 10.1021/acs.bioconjchem.1c00292 (PMC8447191; doi:10.1021/acs.bioconjchem.1c00292)
Supplement: Supplementary file 1 — bc1c00292_si_001.pdf [file bc1c00292_si_001.pdf]

Supporting Information

Peptide Amphiphilic-based Supramolecular Structures with Anti-HIV-1 Activity

Maria J. Gómara[+,*], Ramon Pons[‡], Carolina Herrera[§], Paul Ziprin[#] and Isabel Haro[+,*]

[+] Unit of Synthesis and Biomedical Applications of Peptides. Institute of Advanced Chemistry of Catalonia (IQAC-CSIC). Jordi Girona, 18-26 08034 Barcelona, Spain

[‡] Physical Chemistry of Surfactant Systems. Institute of Advanced Chemistry of Catalonia (IQAC-CSIC). Jordi Girona, 18-26 08034 Barcelona, Spain

[§] Department of Medicine, Imperial College London, London, UK.

[#] Department of Surgery and Cancer, St. Mary's Hospital, Imperial College London, London, UK

*corresponding authors

mariajose.gomara@iqac.csic.es

isabel.haro@iqac.csic.es

Contents:

**Experimental procedures**	S2-S12
**Figure S1:** Primary structure of *N*-peptide amphiphiles	S13
**Figure S2.** Inhibitory activity of peptide derivatives against HIV-1_{BaL} infection	S14
**Figure S3.** Fluorescence emission spectra of W-E1P47 analogues	S15
**Figure S4**. Primary structure of *C*- and *K*-peptide amphiphiles	S16
**Figure S5.** Surface tension (γ)	S17
**Figure S6.** Small Angle X-Ray Scattering	S18
**Figure S7.** Primary structure of the fluorescent lipophilic derivative FAM-C_{18}	S19
**Figure S8.** Fluorescence emission spectra of PAs with POPC LUVs	S20
**Figure S9.** Partitioning isotherms of *C*-PA_{chol}, *K*-PA_{chol} and *N*-, *C*-, *K*-$PA_{mono\text{-}alkyl}$	S21
**Figure S10.** NMR experiments of cholest-5-en-3-yl bromoacetate	S22-S23
**Figure S11.** Dose-response curves obtained in MTT cytotoxicity assays	S24-S25
**Table S1.** Fitting parameters corresponding to the fits of multilayer model in the form of slabs for C-$PA_{mono\text{-}alkyl}$ (A) or polydisperse interacting spheres for N-$PA_{mono\text{-}alkyl}$, N-$PA_{di\text{-}alkyl}$ and K-$PA_{mono\text{-}alkyl}$ (B)	S26
**Table S2.** Fitting parameters corresponding to the fits of Gaussian models	S27
**Table S3.** Fitting parameters of the additional slabs for the asymmetric electronic profiles of PAs dopped POPC vesicles	S28
**Table S4.** Cytotoxic concentration values (CC_{50}) by MTT assay	S29

Experimental procedures

Materials

Solid-phase reactions were performed using a 20 mL syringe that contains a polyethylene filter (Bond Elut, Agilent, CA, USA). NovaSyn TGR resin, 9-fluorenyl-methoxycarbonyl (Fmoc) protected amino acids, 4-(dimethylamino)pyridine (DMAP), 5(6)-carboxyfluoresceine (FAM) and benzotriazole-1-yl-oxy-tris-pyrrolidino-phosphonium hexafluorophosphate (PyBOP) were purchased from Novabiochem (Merck Millipore, Merck KGaA, Darmstadt, Germany). Fmoc-NH-PEG_{27}-COOH and Fmoc-NH-PEG_3-COOH were purchased from Iris Biotech GmbH (Marktredwitz, Germany). Peptide-synthesis-grade dimethylformamide (DMF) and trifluroacetic acid (TFA) were obtained from Scharlau (Barcelona, Spain). HPLC-grade acetonitrile (CH_3CN) was purchased from Fisher Scientific (Loughborough, UK). Acetic and hydrochloric acids were from (Panreac, AppliChem GmbH, Darmstadt, Germany). Diethyl ether, dichloromethane (CH_2Cl_2), and methanol (CH_3OH) were obtained from Merck (KGaA, Darmstadt, Germany). Chloroform ($CHCl_3$) was from Carlo Erba (Val de Reuil, France), Tetrahydrofuran (THF) was from Acros Organics (Geel, Belgium). The coupling reagent, 2-(1H-7-azabenzotriazole-1-yl)-1,1,3,3- tetramethyluronium hexafluorophosphate methanaminium (HATU) was from Genscript (Piscataway, USA). Octadecylamine, dioctadecylamine, cholesterol, succinic anhydride, bromoacetic acid, diisopropylethylamine (DIPEA), triethylamine (Et_3N), *N,N´*-diisopropylcarbodiimide (DIPCDI), 1-hydroxybenzotriazole (HOBt), piperidine, triisopropylsilane (TIS), β-mercaptoethanol, magnesium sulphate ($MgSO_4$) anhydrous and tert-butanol were purchased from Fluka-Sigma-Aldrich (Merck KGaA, Darmstadt, Germany). Chloroform D ($CDCl_3$) was from Euriso-top (St.Aubin Cedez, France).

Thin Layer Chromatography (TLC) were performed on Merck aluminium-backed plates pre-coated with silica (0.2 mm) which were visualized by charring with 5% H_2SO_4 CH_3OH or with phosphomolybdic acid.

NMR experiments of *N*-succinyl-octadecylamine and *N*-succinyl-dioctadecylamine were acquired at 298 K on a 9.4 T Agilent VNMRS spectrometer operating at 400.13 MHz (1H) equipped with a 5 mm OneNMR probe. Chemical shifts (δH) are quoted in parts per million (ppm), referenced to the residual solvent peak as an internal standard. Coupling constants (J) are reported to the nearest 0.1 Hz.

NMR experiments of cholest-5-en-3-yl bromoacetate were acquired at 298 K on a 11.7 T Bruker AVANCE IIIHD spectrometer operating at 500.13 MHz (1H) equipped with a 5 mm cryogenically cooled triple-resonance probehead (TCI).

Cysteinyl-PEG_X-peptides were purified by HPLC at semipreparative scale on a Waters 1525P with a UV detector Waters 2489 (Waters Corporation, Mildford, USA) using an Agilent ZORBAX SB-C_{18} (semi-preparative RP, 9.4x250 mm, particle size 5 µm) (Agilent Technologies, Santa Clara, USA). Pure peptides were characterized by UPLC-MS on Waters ACQUITY UPLC (Waters Corporation, Mildford, USA) with the column ACQUITY UPLC BEH C_{18} (RP, 2.1 x 100 µm, particle size 1.7 µm) with both detector UV-Vis and an electrospray ionization mass spectrometry (ESI-MS) Waters LCT Premier XE (Micromass Waters, Milford, MA, USA).

PAs were purified by Flash chromatography (FC) on Isolera One Biotage (Biotage AB, Uppsala, Sweden) with the cartridge Biotage SNAP Bio HP-Biosphere (reverse phase (RP) C_4 12g, pore size 300 Å, particle size 20 µm). Purified products were characterized by MS performed by Flow-Injection analysis (FIA) on HPLC Waters 2795 Alliance with a detector DAD Agilent 1100 and MS detector ESI triple quadrupole Quattro Micro Waters (Micromass Waters, Milford, MA, USA).

Lyophilization was performed on a Christal Alpha 2-4 LD Plus freeze dryer (Martin Christ GmbH, Osterode am Harz, Germany). Evaporation in vacuo was performed on a Heidolph Laborota 4001 efficient (Heidolph Instruments GmbH & CO, Schwabach, Germany). Purified products were weighted on an analytical microbalance Mettler Toledo XPR2 (Mettler-Toledo GmbH, Greifensee, Switzerland).

Synthesis of $PA1_{PEG3}$

The Fmoc deprotected E1P47 on solid support (0.200 g, 0.0258 mmol, 1.0 equiv) was solvated with DMF overnight. After removing the solvent, a mixture of Fmoc-NH-$(PEG)_3$-COOH (0.025 g, 0.0516 mmol, 2.0 equiv), PyBOP (0.027 g, 0.0516 mmol, 2.0 equiv) and HOBt (0.007 g, 0.0516 mmol, 2.0 equiv) dissolved in the minimum amount of DMF was added to the peptidyl-resin. Then, DIEA (0.018 mL, 0.103 mmol, 4 equiv) was added and was left reacting overnight with occasional stirring.
After removing the Fmoc group by treatment with 20% (v/v) piperidine in DMF, a mixture of *N*-succinyl octadecylamine (0.028 g, 0.0774 mmol, 3.0 equiv), PyBOP (0.040 g, 0.0774 mmol, 3.0 equiv), HOBt (0.010 g, 0.0774 mmol, 3.0 equiv) and DIEA (0.027 mL, 0.1548 mmol, 6.0 equiv) dissolved in the minimum amount of CH_2Cl_2/DMF 70:30 (v/v) was added to the peptidyl-resin in the same reactor at room temperature

with occasional stirring overnight. The acylation and deprotection reactions were checked by the ninhydrin test.

The cleavage and deprotection of the dried peptidyl-resin was effected by treatment with 20 mL of a mixture 95% TFA (v/v), H_2O 2.5% (v/v) and 2.5% TIS (v/v) for 6 hours with occasional stirring at room temperature. The TFA was evaporated with N_2 flow. Cold water was added to precipitate the crude peptide that was isolated by centrifugation (4000 rpm; 5 °C; 10 minutes). The precipitate was dissolved in acetic acid 10%, frozen in a dry ice / acetone bath (-78 ° C) and lyophilized (0.049 g, yield (synthesis): 63%). The crude peptide was purified by FC-UV/Vis ($\lambda_{collection}$ = 220 nm, $\lambda_{tracking}$ = 280 nm) Isolera one Biotage with the cartridge Biotage SNAP Bio HP-Biosphere (reverse phase C4 12g, pore size 300 Å, particle size 20 µm) eluted with CH_3CN + 0.05% TFA and H_2O + 0.05% TFA at a flow rate of 10 ml/min. Gradient: 30% (CH_3CN) 3.0 column volume (CV), 30%-100% (CH_3CN) 6.0 CV, 100% (CH_3CN) 3.0 CV. The compound was released at 83% CH_3CN (7.6 CV t_R=11.4min). Finally, the final product was characterized by mass spectroscopy (MS) performed by FIA with injector of electrospray ionization (ESI) (vol. injection = 20 µL, concentration = 0.5 mg/mL). The possible mass peaks were predicted by MassLynx V4.1 software. LRMS: calculated m/z for $C_{155}H_{230}N_{28}O_{30}$ 1483.83 $[M+2H]^{2+}$, 989.56 $[M+3H]^{3+}$; found by MS (ESI) 1483.71 $[M+2H]^{2+}$, 989.65 $[M+3H]^{3+}$.

The PA1$_{PEG3}$ primary structure is shown in **Figure S1**. White salt (MW 2965.65) was obtained (0.021 g, yield (synthesis + purification): 27%).

Synthesis of PA1$_{PEG27}$ (*N*-PA$_{mono\text{-}alkyl}$)

Fmoc deprotected E1P47 on solid support (0.558 g, 0.072 mmol, 1.0 equiv.) was solvated overnight with DMF. Then, the solvent was removed and a mixture of Fmoc-NH-$(PEG)_{27}$-COOH (0.222 g, 0.144 mmol, 2 equiv.), PyBOP (0.075 g, 0.144 mmol, 2 equiv.), HOBt (0.019 g, 0.144 mmol, 2 equiv.) and DIEA (0.050 mL, 0.288 mmol, 4 equiv.) solved in the minimum amount of DMF were added overnight. After deprotection of Fmoc, *N*-succinyl-octadecylamine (0.080 g, 0.216 mmol, 3.0 equiv.), PyBOP (0.112 g, 0.216 mmol, 3 equiv.), HOBt (0.029 g, 0.216 mmol, 3 equiv.) and DIEA (0.075 mL, 0.432 mmol, 6 equiv.) were solved in the minimum amount of CH_2Cl_2/DMF 70/30 (v/v) and added to the pegilated peptidyl-resin in the same reactor at room temperature with occasional stirring overnight. After drying the peptidyl-resin, a cleavage was effected by treatment with 20 mL 95% (v/v) TFA, 2.5% (v/v) H_2O and 2.5% (v/v) TIS for 3h at room temperature. The TFA was evaporated with N_2 flow.

Finally, water was added, frozen with dry ice/acetone bath (-78 ºC) and lyophilized overnight.

The crude peptide was purified by FC-UV/Vis using the same equipment and column described for $PA1_{PEG3}$ at a flow rate of 12ml/min. Gradient: 40%-50% (CH_3CN) 1.0 column volume (CV), 50%-60% 3.0 CV, 60%-100% 9 CV, 100% 2 CV. The compound was released at 70% CH_3CN (t_R=8 min). The final product was solved in CH_3CN/H_2O 40:60 and was confirmed by FIA-ESI-MS. The possible mass peaks were predicted by MassLynx V4.1 software. LRMS: calculated m/z for $C_{203}H_{326}N_{28}O_{54}$: 1341.98 $[M+3H]^{3+}$, 1349.31 $[M+Na]^{3+}$, 1006.74 $[M+4H]^{4+}$, 1016.26 $[M+K]^{4+}$; found by MS (ESI): 1341.95 $[M+3H]^{3+}$, 1349.27 $[M+Na]^{3+}$, 1006.81 $[M+4H]^{4+}$, 1016.30 $[M+K]^{4+}$.

The $PA1_{PEG27}$ primary structure shown in **Figure S1**. White salt (MW 4022.91) was obtained (0.0326 g, yield (synthesis + purification): 11%).

Synthesis of $PA2_{PEG3}$

A mixture of Fmoc-NH-$(PEG)_3$-COOH (0.019 g, 0.039 mmol, 2.0 equiv), PyBOP (0.020 g, 0.038 mmol, 2.0 equiv) and HOBt (0.005 g, 0.037 mmol, 2.0 equiv) and DIEA (0.013 mL, 0.0746 mmol, 4 equiv) dissolved in the minimum amount of DMF was added to a fraction of E1P47 peptidyl-resin (0.15 g, 0.0193 mmol, 1.0 equiv). After deprotection of Fmoc, a mixture of *N*-succinyl-dioctadecylamine (0.034 g, 0.057 mmol, 3.0 equiv.), PyBOP (0.030 g, 0.0576 mmol, 3.0 equiv.), HOBt (0.008 g, 0.059 mmol, 3.0 equiv.) and DIEA (0.020 mL, 0.1148 mmol, 6.0 equiv.) dissolved in the minimum amount of CH_2Cl_2:DMF 70:30 was added to the peptidyl-resin in the same reactor at room temperature with occasional stirring overnight.

The cleavage was performed following the same procedure detailed for $PA1_{PEG3}$ (0.042 g, 68% cleavage yield). The crude peptide was purified by FC-UV/Vis using the same equipment and column described for $PA1_{PEG3}$. The gradient used was 30% (CH_3CN) 3.0 CV, 30%-100% (CH_3CN) 6.0 CV, 100% (CH_3CN) 6.0 CV. The compound was released at 100% CH_3CN (9.5 CV t_R=14.2). The final product was confirmed by FIA-ESI-MS. LRMS: calculated m/z for $C_{173}H_{266}N_{28}O_{30}$; 1610.07 $[M+2H]^{2+}$, 1073.72 $[M+3H]^{3+}$; found by MS (ESI): 1610.02 $[M+2H]^{2+}$, 1073.68 $[M+3H]^{3+}$.

The $PA2_{PEG3}$ primary structure is shown in **Figure S1.** White salt (MW 3218.13) was obtained (0.010 g, yield (synthesis + purification): 16%)

Synthesis of $PA2_{PEG27}$ (*N*-$PA_{di\text{-}alkyl}$)

Fmoc-NH-$(PEG)_{27}$-COOH was coupled to a fraction of E1P47 peptidyl-resin (0.558 g, 0.072 mmol, 1.0 equiv.) following the same procedure described for $PA1_{PEG27}$. After

deprotection of Fmoc, *N*-succinyl-dioctadecylamine (0.128 g, 0.216 mmol, 3.0 equiv), PyBOP (0.112 g, 0.215 mmol, 3 equiv), HOBt (0.029 g, 0.215 mmol, 3 equiv) and DIEA (0.075 mL, 0.430 mmol, 6 equiv) solved in the minimum amount of CH_2Cl_2/DMF 70/30 (v/v) were added to the pegilated peptidyl-resin in the same reactor at room temperature with occasional stirring overnight. Once the synthesis was completed, the cleavage reaction was carried out using the same conditions described for $PA1_{PEG27}$. Finally, PA was solved with AcOH 30%, frozen with dry ice/acetone bath (-78 ºC) and lyophilized overnight.

The crude peptide was purified by FC-UV/Vis using the same equipment and column described for $PA1_{PEG3}$ at a flow rate of 12 mL/min. Gradient: 40%-50% (CH_3CN) 2.0 CV, 50%-60% 5.0 CV, 60%-100% 2 CV, 100% 2 CV. The compound was released at 100% CH_3CN (t_R: 13 min). The product was solved in CH_3CN/H_2O 40:60 and the final product was confirmed by FIA-ESI-MS. LRMS: calculated m/z for $C_{221}H_{362}N_{28}O_{54}$ 1426.14 $[M+3H]^{3+}$, 1069.86 $[M+4H]^{4+}$, 863.91 $[M+K]^{5+}$; found by MS (ESI): 1425.94 $[M+3H]^{3+}$, 1069.80 $[M+4H]^{4+}$, 863.69 $[M+K]^{5+}$.

The $PA2_{PEG27}$ primary structure is shown in **Figure S1**. White crystals (MW 4275.39) were obtained (0.044 g, yield (synthesis + purification): 14%).

Synthesis of $PA3_{PEG3}$

Fmoc-NH-$(PEG)_3$-COOH was coupled to a fraction of E1P47 peptidyl-resin (0.15 g, 0.019 mmol, 1.0 equiv) following the same procedure described for $PA2_{PEG3}$. Upon deprotection of Fmoc group, a mixture of Fmoc-NH-Cys(trt)-COOH (0.034 g, 0.058 mmol, 3.0 equiv), PyBOP (0.030 g, 0.058 mmol, 3.0 equiv), HOBt (0.008 g, 0.059 mmol, 3.0 equiv) and DIEA (0.020mL, 0.115 mmol, 6.0 equiv) were dissolved in the minimum amount of CH_2Cl_2:DMF (70:30) and added to the pegylated peptidyl-resin. The reaction took place for 45 min at room temperature.

After deprotecting the Fmoc, the peptidyl-resin was dried and the cleavage reaction was done by treatment with a mixture of 20mL of 94% (v/v) TFA, 2.5% (v/v) H_2O, 2.5% (v/v) β-mercaptoethanol and 1% (v/v) TIS for 6 hours with occasional stirring at room temperature. After collecting the filtrate, the TFA was evaporated by N_2 flow. The crude peptide was precipitated by adding cold diethyl ether and isolated by centrifugation (4000 rpm; 5°C; 10 minutes). The precipitate was dissolved in acetic acid 10% (v/v), frozen in a dry ice/acetone bath (-78°C) and lyophilized. The crude peptide was purified by RP-HPLC at semipreparative scale using an Agilent ZORBAX SB-C18 (semi-preparative RP, 9.4x250 mm, particle size 5 µm) column. The purification was carried out at a flow rate of 8 mL/min using a detection wavelength of 220nm. A linear gradient 10%-95% CH_3CN/H_2O + 0.05% TFA in 30 min was used. The purified product was

confirmed by UPLC-MS t_R (G 5-100% CH_3CN/H_2O in 10 min): 3.4 min. LRMS: calculated m/z for $C_{136}H_{194}N_{28}O_{29}S$ 1359.62 $[M+2H]^{2+}$, 906.75 $[M+3H]^{3+}$ found by MS (ESI): 1359.23 $[M+2H]^{2+}$, 906.49 $[M+3H]^{3+}$. A white product was obtained (0.018 g, yield (synthesis + purification): 35%).

Finally, $PA3_{PEG3}$ was obtained by conjugation of the cystenyl-PEG_3-peptide and the synthesized cholesterol derivative. Cys-PEG_3-E1P47 (0.018 g, 0.007 mmol, 1.0 equiv) was dissolved in 1.4 mL of DMSO and cholest-5-en-3-yl-bromoacetate (0.004 g, 0.008 mmol, 1.2 equiv) dissolved in 0.68 mL of THF was added. Then, 1% (v/v) of DIEA (0.021 mL) was added to the solution and was left reacting with stirring for 3:30 h at room temperature. Afterwards, 10 mL H_2O and 20 mL tert-butanol were added to the solution, frozen with a dry ice/acetone bath (-78°C) and lyophilized. The lyophilization process was repeated several times until obtaining white crystals (MW 3143.91) (0.018 g, yield 28%). The final product was confirmed by FIA-ESI-MS. LRMS: calculated m/z for $C_{165}H_{240}N_{28}O_{31}S$ 1048.98 $[M+3H]^{3+}$, 796.76 $[M+K]^{4+}$; found by MS (ESI): 1048.95 $[M+3H]^{3+}$, 796.04 $[M+K]^{4+}$

The $PA3_{PEG3}$ primary structure is shown in **Figure S1**.

Synthesis of $PA3_{PEG27}$ (*N*-PA_{chol})

A mixture of Fmoc-NH-$(PEG)_{27}$-COOH (0.0865 g, 0.056 mmol, 2 equiv.) activated with PyBOP (0.029 g, 0.056 mmol, 2 equiv.), HOBt (0.008 g, 0.059 mmol, 2 equiv.) and DIEA (0.020 mL, 0.115 mmol, 4 equiv.) solved in the minimum amount of DMF was added to a fraction of Fmoc-deprotected E1P47 peptidyl-resin (0.217 g, 0.028 mmol, 1.0 equiv.). The reaction took place overnight at room temperature. Afterwards, Fmoc-Cys(trt)-OH (0.049 g, 0.084 mmol, 3 equiv.), HATU (0.032 g, 0.084 mmol, 3 equiv.) and DIEA (0.029 mL, 0.166 mmol, 6 equiv.) were solved in the minimal amount of DMF and added to the Fmoc deprotected peptidyl-resin. The reaction took place for 45 min at room temperature. After deprotecting the Fmoc, the peptidyl-resin was dried and the cleavage reaction was done by treatment with 94% (v/v) TFA, 2.5% (v/v) H_2O, 2,5% (v/v) β-mercaptoethanol and 1% (v/v) TIS. The reaction took place for 3 hours at room temperature. After collecting the filtrate, the TFA was removed by N_2 flow and the crude peptide was precipitated with cold diethylether. The isolation of the peptide was done as described for $PA3_{PEG3}$.

The crude peptide was purified by RP-HPLC at semipreparative scale using the same condition described for $PA3_{PEG3}$. The purified product was confirmed by UPLC-MS t_R (G 5-100% CH_3CN/H_2O in 10 min): 3.1 min. LRMS: calculated m/z for $C_{184}H_{290}N_{28}O_{53}S$ 1259.17 $[M+3H]^{3+}$, 944.63 $[M+4H]^{4+}$; found by MS (ESI): 1258.93 $[M+3H]^{3+}$, 944.36

$[M+4H]^{4+}$. White crystals were obtained (0.0112 g, yield (synthesis + purification): 10.6%).

Purified Cys-PEG_{27}-E1P47 (0.011 g, 0.0029 mmol, 1 equiv) was dissolved in 0.6 mL DMSO and cholest-5-en-3-yl bromoacetate (0.0018 g, 0.0035 mmol, 1.2 equiv.) dissolved in 0.3 mL THF was added. Then 1% by volume (0.009 mL) of DIEA was added to the solution. The reaction took place for 3 h at room temperature. Afterwards, 10 mL H_2O and 20 mL tert-butanol were added to the solution, frozen with a dry ice/acetone bath (-78°C) and lyophilized. The lyophilization process was repeated several times until obtaining white crystals (MW 4201.17) (0.011 g, yield 9.6%). The final product was confirmed by FIA-ESI-MS. LRMS: calculated m/z for $C_{213}H_{336}N_{28}O_{55}S$ 1401.40 $[M+3H]^{3+}$, 1051.30 $[M+4H]^{4+}$; found by MS (ESI): 1401.15 $[M+3H]^{3+}$, 1051.12 $[M+4H]^{4+}$

The $PA3_{PEG27}$ primary structure is shown in **Figure S1**.

Synthesis of *C*-PA$_{\text{mono-alkyl}}$

As described for *N*-PA$_{\text{mono-alkyl}}$, *N*-succinyl-octadecylamine (0.031 g, 0.084 mmol, 3 equiv), PyBOP (0.044 g, 0.084 mmol, 3 equiv), HOBt (0.011 g, 0.084 mmol, 3 equiv) and DIEA (0.029 mL, 0.168 mmol, 6 equiv) solved in the minimum amount of CH_2Cl_2/DMF 60/40 (v/v) were added to the pegilated peptidyl-resin and was left overnight at room temperature. Once the synthesis was completed, the cleavage reaction was carried out by treatment with TFA 95% (v/v), TIS 2.5% (v/v) and H_2O 2.5% (v/v) for 6 h. Afterwards, TFA was evaporated with N_2 flow and the peptide was precipitated with cold water. The precipitate was isolated after centrifugation at 4000 rpm at 4ºC for 10 min, solved in H_2O/CH_3CN (50:50) and lyophilized.

The crude peptide was purified by FC–UV/Vis with the cartridge Biotage SNAP Bio HP-Biosphere (reverse phase C4 12g, pore size 300 Å, particle size 20 µm) eluted with CH_3CN + 0.05% TFA and H2O + 0.05% TFA at a flow rate of 10 mL/min. Gradient: 40% (CH_3CN) 4.0 column volume (CV), 40%-100% (CH_3CN) 8.0 CV, 100% 4 CV. The compound was eluted at 65% of CH_3CN (t_R:11 min). The final product was confirmed by FIA-ESI-MS. LRMS: calculated m/z for $C_{209}H_{338}N_{30}O_{55}$ 1384.70 $[M+3H]^{3+}$, 1038.78 $[M+4H]^{4+}$. found by MS (ESI): 1384.64 M^{3+}, 1038.65 $[M+4H]^{4+}$

The *C*-PA$_{\text{mono-alkyl}}$ primary structure is shown in **Figure S1**. White crystals (MW 4151.09) (0.027 g, yield (synthesis + purification): 23% yield).

Synthesis of *C*-PA$_{\text{di-alkyl}}$

As described for *N*-PA$_{\text{di-alkyl}}$, *N*-succinyl-dioctadecylamine (0.050 g, 0. 084 mmol, 3 equiv), PyBOP (0.044 g, 0.084 mmol, 3 equiv), HOBt (0.011 g, 0.084 mmol, 3 equiv)

and DIEA (0.029 mL, 0.168 mmol, 6 equiv) solved in the minimum amount of CH_2Cl_2/DMF 60/40 (v/v) were added to the pegilated peptidyl-resin and the reaction took place overnight.

The peptide was isolated and purified as described for *C*-$PA_{mono-alkyl}$. The compound was released at 85% of CH_3CN (t_R =15 min). The purified product was analyzed ESI-MS. LRMS: calculated m/z for $C_{227}H_{374}N_{30}O_{55}$: 1468.86 $[M+3H]^{3+}$, 1101.90 $[M+4H]^{4+}$, 881.72 $[M+5H]^{5+}$, 734.94 $[M+6H]^{6+}$; found by MS (ESI): 1468.85 $[M+3H]^{3+}$, 1101.82 $[M+4H]^{4+}$, 881.52 $[M+5H]^{5+}$, 734.72 $[M+6H]^{6+}$

The *C*-$PA_{di-alkyl}$ primary structure is shown in **Figure S1**. White crystals (MW 4403.57) (0.026 g, yield (synthesis + purification): 21%).

Synthesis of *C*-PA_{chol}

After coupling the PEG_{27} moiety, Fmoc-Cys(trt)-OH (0.049 g, 0.084 mmol, 3 equiv), PyBOP (0.044 g, 0.084 mmol, 3 equiv), HOBt (0.011 g, 0.084 mmol, 3 equiv) and DIEA (0.029 mL, 0.168 mmol, 6 equiv) were coupled to the peptidyl-resin at room temperature overnight. The cleavage procedure and the subsequent purification of the crude peptide were performed as described for *N*-PA_{chol}. A linear gradient 15%-95% CH_3CN/H_2O + 0.05% TFA in 30 min was used for peptide purification. The purified product was confirmed by UPLC-MS t_R (G 5-100% CH_3CN/H_2O in 10 min): 3.1 min. LRMS: calculated m/z for $C_{190}N_{302}N_{30}O_{54}S$ 1301.90 $[M+3H]^{3+}$, 976.67 $[M+3H]^{4+}$; found by MS (ESI): 1301.73 $[M+3H]^{3+}$, 976.55 $[M+3H]^{4+}$. White crystals were obtained (0.011 g, yield (synthesis+ purification 10%).

Cys-PEG_{27}-E1P47 (0.011 g, 0.0028 mmol, 1 equiv) was dissolved in 0.6 mL DMSO and cholest-5-en-3-yl bromoacetate (0.0017 g, 0.0034 mmol, 1.2 equiv) dissolved in 0.3 mL THF was added. Then 1% by volume (0.010 mL) of DIEA was added to the solution. The reaction took place for 3.5 h at room temperature. The solution was lyophilized as described for the *N*-PA_{chol}. White crystals (MW 4329.34) were obtained (0.012 g, yield 9.2%). The final product was confirmed by FIA-ESI-MS. LRMS: calculated m/z for $C_{219}H_{348}N_{30}O_{56}S$; 1044.12 $[M+3H]^{3+}$, 1083.34 $[M+4H]^{4+}$, 866.87 $[M+5H]^{5+}$; found by MS (ESI): 1444.97 $[M+3H]^{3}$+, 1083.33 $[M+4H]^{4}$+, 867.41 $[M+5H]^{5+}$

The *C*-PA_{chol} primary structure is shown in **Figure S1**.

Synthesis of *K*-$PA_{mono-alkyl}$

As described for *N*-$PA_{mono-alkyl}$ and *C*-$PA_{mono-alkyl}$, *N*-succinyl-octadecylamine (0.029 g, 0.078 mmol, 3 equiv), PyBOP (0.041 g, 0.079 mmol, 3 equiv), HOBt (0.011 g, 0.081 mmol, 3 equiv) and DIEA (0.027 mL, 0.155 mmol, 6 equiv) solved in the minimum

amount of CH_2Cl_2/DMF 60/40 (v/v) were added to the pegilated peptidyl-resin and was left overnight at room temperature. Once the synthesis was completed, the cleavage reaction was carried out and the peptide was isolated as described for *C*-PA$_{mono\text{-}alkyl}$.

The crude peptide was purified by FC–UV/Vis using the same elution conditions as for *C*-PA$_{mono\text{-}alkyl}$. The compound was eluted at 70% of CH_3CN (t_R=12 min). The final product was confirmed by FIA-ESI-MS. LRMS: calculated m/z for $C_{203}H_{326}N_{28}O_{54}$: 1341.98 $[M+3H]^{3+}$, 1006.74 $[M+4H]^{4+}$; found by MS (ESI): 1341.80 $[M+3H]^{3+}$, 1006.59 $[M+4H]^{4+}$

The *K*-PA$_{mono\text{-}alkyl}$ primary structure is shown in **Figure S1.** White crystals (MW 4022.91) (0.031 g, yield (synthesis+purification): 30% yield).

Synthesis of *K*-PA$_{di\text{-}alkyl}$

As described for *N*-PA$_{di\text{-}alkyl}$ and *C*-PA$_{di\text{-}alkyl}$, *N*-succinyl-dioctadecylamine (0.046 g, 0.078 mmol, 3 equiv), PyBOP (0.041 g, 0.079 mmol, 3 equiv), HOBt (0.011 g, 0.081 mmol, 3 equiv) and DIEA (0.028 mL, 0.161 mmol, 6 equiv) solved in the minimum amount of CH_2Cl_2/DMF 60/40 (v/v) (~5 mL) were added to the pegilated peptidyl-resin and the reaction took place overnight.

The peptide isolation and subsequent purification was done as described for *K*-PA$_{mono\text{-}alkyl}$. The compound was released at 85% of CH_3CN (t_R=15 min). The purified product was analysed by mass spectroscopy (ESI-MS). LRMS: calculated m/z for $C_{221}H_{362}N_{28}O_{54}$: 1426.14 $[M+3H]^{3+}$, 1069.86 $[M+4H]^{4+}$; found by MS (ESI): 1425.94 $[M+3H]^{3+}$, 1069.83 $[M+4H]^{4+}$

The *K*-PA$_{di\text{-}alkyl}$ primary structure is shown in **Figure S1**. White product (MW 4275.39) (0.027 g, yield (synthesis + purification): 24%).

Synthesis of *K*-PA$_{chol}$

After coupling the PEG moiety, Fmoc-Cys(trt)-OH (0.045 g, 0.077 mmol, 3 equiv), HATU (0.030 g, 0.079 mmol, 3 equiv) and DIEA (0.027 mL, 0.155 mmol, 6 equiv) were coupled to the peptidyl-resin for 45 min at room temperature. The cleavage procedure and the subsequent purification of the crude peptide were performed as described for *N-PA$_{chol}$*. The purified product was confirmed by UPLC-MS t_R (G 5-100% CH_3CN/H_2O in 10 min): 3.1 min. LRMS: calculated m/z for $C_{184}H_{290}N_{28}O_{53}S$ 1259.17 $[M+3H]^{3+}$, 944.63 $[M+4H]^{4+}$; 755.91 $[M+5H]^{5+}$; found by MS (ESI): 1259.00 $[M+3H]^{3+}$, 944.36 $[M+4H]^{4+}$, 755.57 $[M+5H]^{5+}$. White crystals were obtained (0.0094 g, yield (synthesis + purification): 9.6%).

Cys-PEG$_{27}$-E1P47 (0.0094 g, 0.0025 mmol, 1 equiv) was dissolved in 0.6 mL DMSO and cholest-5-en-3-yl bromoacetate (0.0015 g, 0.003 mmol, 1.2 equiv) dissolved in 0.3 mL THF was added. Then 1% by volume (0.009 mL) of DIEA was added to the solution. The reaction took place for 3 h at room temperature. The solution was lyophilized as described for the *N-PA$_{chol}$*. White crystals (MW 4201.17) were obtained (0.010 g, yield 9.2%). The final product was confirmed by FIA-ESI-MS. LRMS: calculated m/z for $C_{213}H_{336}N_{28}O_{55}S$: 1401.40 $[M+3H]^{3+}$, 1051.30 $[M+4H]^{4+}$. Found: 1401.16 $[M+3H]^{3+}$, 1051.07 $[M+4H]^{4+}$

The *K*-PA$_{chol}$ primary structure is shown in **Figure S1**.

Synthesis of a FAM-C$_{18}$

The synthesis of the fluorescent lipophilic derivative, FAM-C$_{18}$, was carried out on a Rink-amide (MBHA) resin (0.271 g, 0.73 mmol.g^{-1}, 0.198 mmol). After swelling the resin overnight with DMF, Fmoc group was deprotected by treatment twice with 20% (v/v) piperidine in DMF for 10 min. An Fmoc-Lys(Mtt)-OH derivative (0.371 g, 0.594 mmol, 3 equiv) was coupled to the resin by activation with PyBOP (0.309 g, 0.594 mmol, 3 equiv), HOBt (0.080 g, 0.594 mmol, 3 equiv) and DIEA (0.207 mL, 1.188 mmol, 6 equiv) in DMF. The reaction took place overnight at room temperature. After deprotection of Fmoc, stearic acid (0.169 g, 0.594 mmol, 3 equiv) was coupled to the lysine *Nα* by activation with PyBOP (3 equiv), HOBt (3 equiv) and DIEA (6 equiv) in CH_2Cl_2/DMF 60/40 (v/v). The reaction took place overnight and was repeated twice. After washing the resin x5 with CH_2Cl_2, the deprotection of Mtt group was carried out by treatment 5x for 15 min with a solution of CH_2Cl_2 95% (v/v), TIS 4% (v/v) and TFA 1% (v/v). Then, the resin was rinsed x5 with DMF and 5(6)-carboxyfluorescein (FAM) (0.223 g, 0.593 mmol, 3 equiv) was coupled twice to the lysine *Nε* by activation with PyBOP (3 equiv), HOBt (3 equiv) and DIEA (6 equiv). The reaction took place overnight. All coupling and deprotection steps were checked by the Kaiser test.

Once the synthesis was completed, a cleavage reaction of the third part of the resin was carried out by treatment with TFA 95% (v/v), TIS 2.5% (v/v) and H_2O 2.5% (v/v) for 3 h. Afterwards, TFA was evaporated with N_2 flow and the peptide was precipitated with cold water. The precipitate was isolated after centrifugation at 4000 rpm at 4ºC for 10 min, solved in tert-butanol and lyophilized.

The crude fluorescent derivative (0.030 g) was purified by FC–UV/Vis with the cartridge Biotage SNAP Bio HP-Biosphere (reverse phase C4 12g, pore size 300 Å, particle size 20 µm) eluted with CH_3CN + 0.05% TFA and H_2O + 0.05% TFA at a flow rate of 10 mL/min. Gradient: 30% (CH_3CN) 3.0 column volume (CV), 30%-85% (CH_3CN) 6.0 CV. The compound was eluted at 76% of CH_3CN (t_R=12 min). The final product was

confirmed by ESI-MS. LRMS: calculated m/z for $C_{45}H_{59}N_3O_8$; 770.97 $[M+1H]^{1+}$; found by MS (ESI): 770.67 $[M+1H]^{1+}$

The FAM-C_{18} primary structure and is shown in **Figure S1.** Orange salt (MW 769.98) (0.006 g, yield (purification): 20%).

N-peptide amphiphiles

$PA1_{PEG3}$

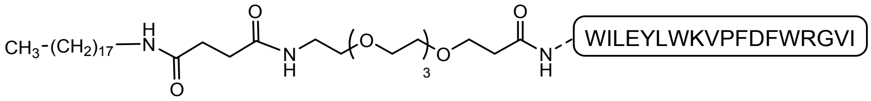

$PA1_{PEG27}$ (N-$PA_{mono\text{-}alkyl}$)

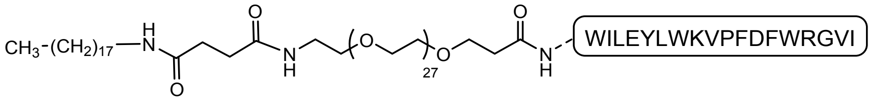

$PA2_{PEG3}$

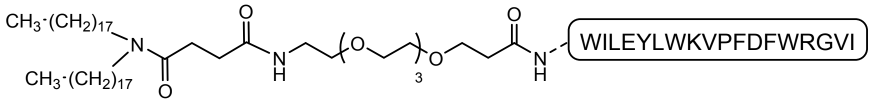

$PA2_{PEG27}$ (N-$PA_{di\text{-}alkyl}$)

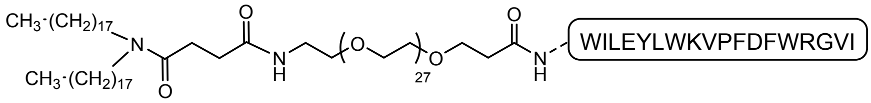

$PA3_{PEG3}$

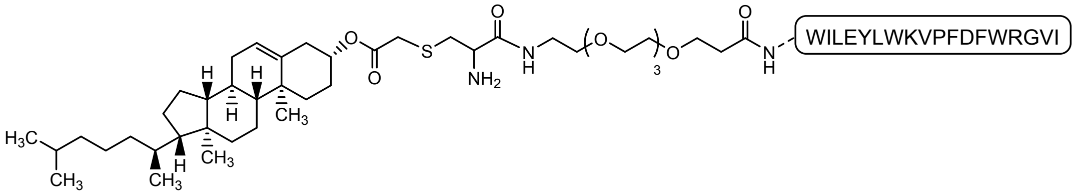

$PA3_{PEG27}$ (N-PA_{chol})

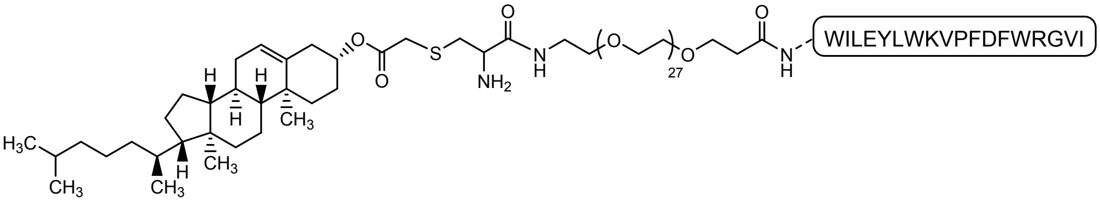

Figure S1. Primary structure of *N*-peptide amphiphiles

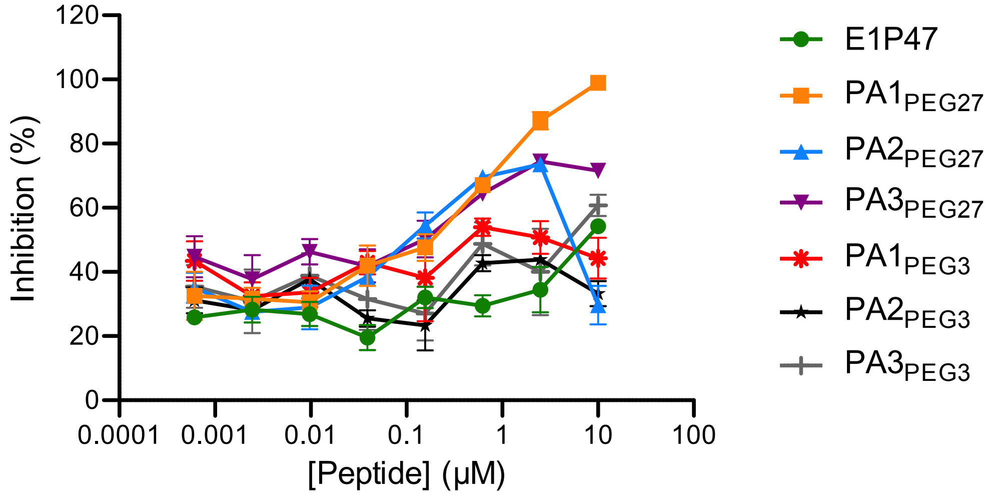

Figure S2. Inhibitory activity of E1P47 peptide derivatives against HIV-1_{BaL} infection. TZM-bl cells were treated for 1 h in the presence or absence of serial dilutions of peptides prior to addition of HIV-1_{BaL}. Luciferase expression in TZM-bl cells (measured in relative light units) was determined after 48 h of culture and the extent of inhibition by each drug was calculated. The percentage of inhibition was normalized relative to the relative light units obtained for TZM-bl cells not exposed to virus (0% infectivity) and for cells infected with virus in the absence of compound (100% infectivity). Data are the means (± SEM) from triplicates.

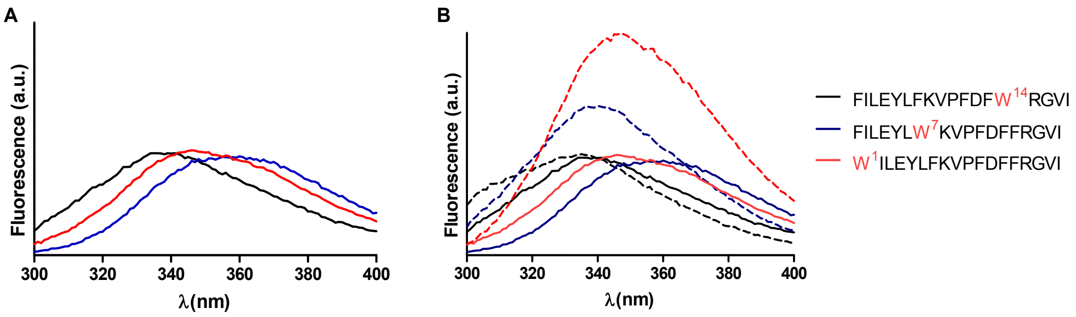

Figure S3. (A) Fluorescence emission spectra of W^{1}-E1P47, W^{7}-E1P47 and W^{14}-E1P47 analogues (peptide concentration 5.0 x 10^{-6} M in HEPES buffer (0.01 M, pH 7.4); **(B)** Fluorescence emission spectra of the peptides in aqueous media (solid line) and in presence of POPC LUVs at a peptide ratio of 1:100 (dashed line).

C-peptide amphiphiles

C-$PA_{mono\text{-}alkyl}$

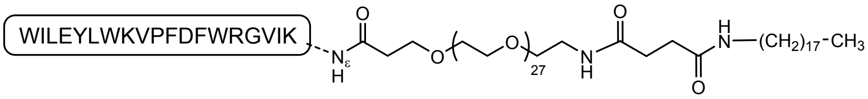

C-$PA_{di\text{-}alkyl}$

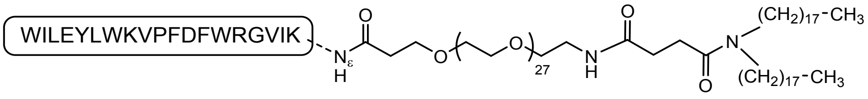

C-PA_{chol}

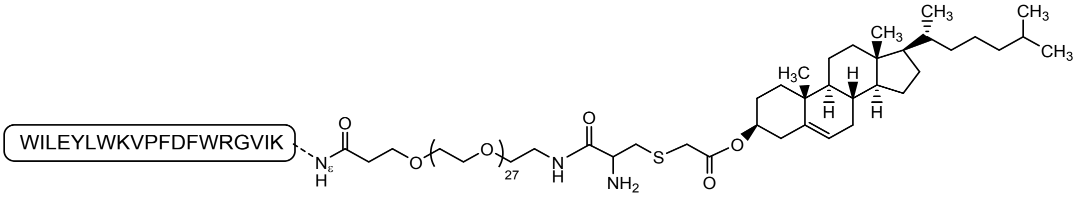

K-peptide amphiphiles

K-$PA_{mono\text{-}alkyl}$

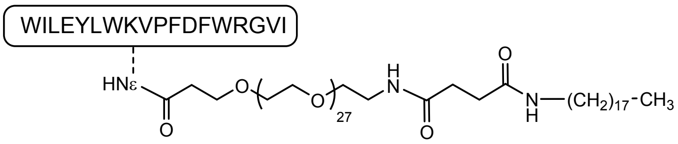

K-$PA_{di\text{-}alkyl}$

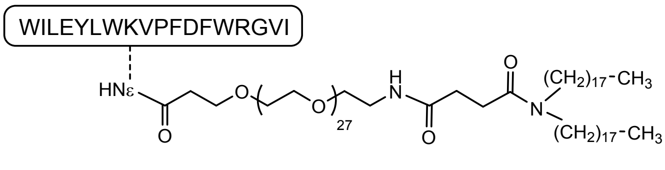

K-PA_{chol}

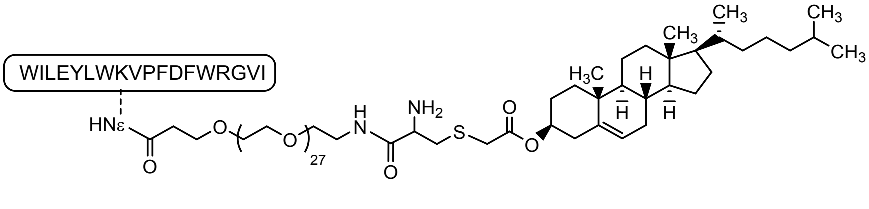

Figure S4. Primary structure of *C*- and *K*-peptide amphiphiles

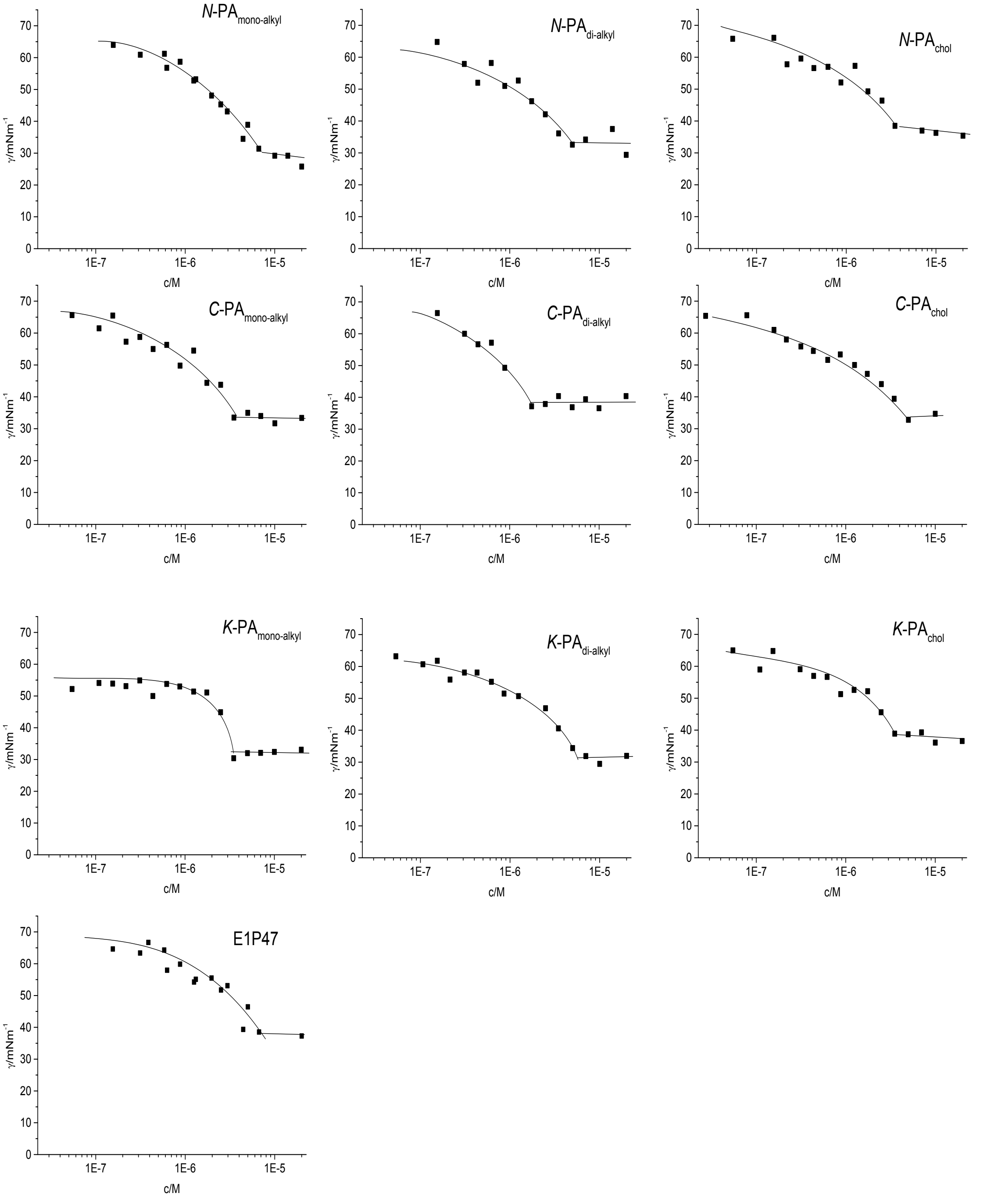

Figure S5. Surface tension (γ) as a function of concentration in DMSO 0.5% measured at 25ºC. The lines are a guide for the eyes. The critical concentration is taken at the point where the surface tension stabilizes.

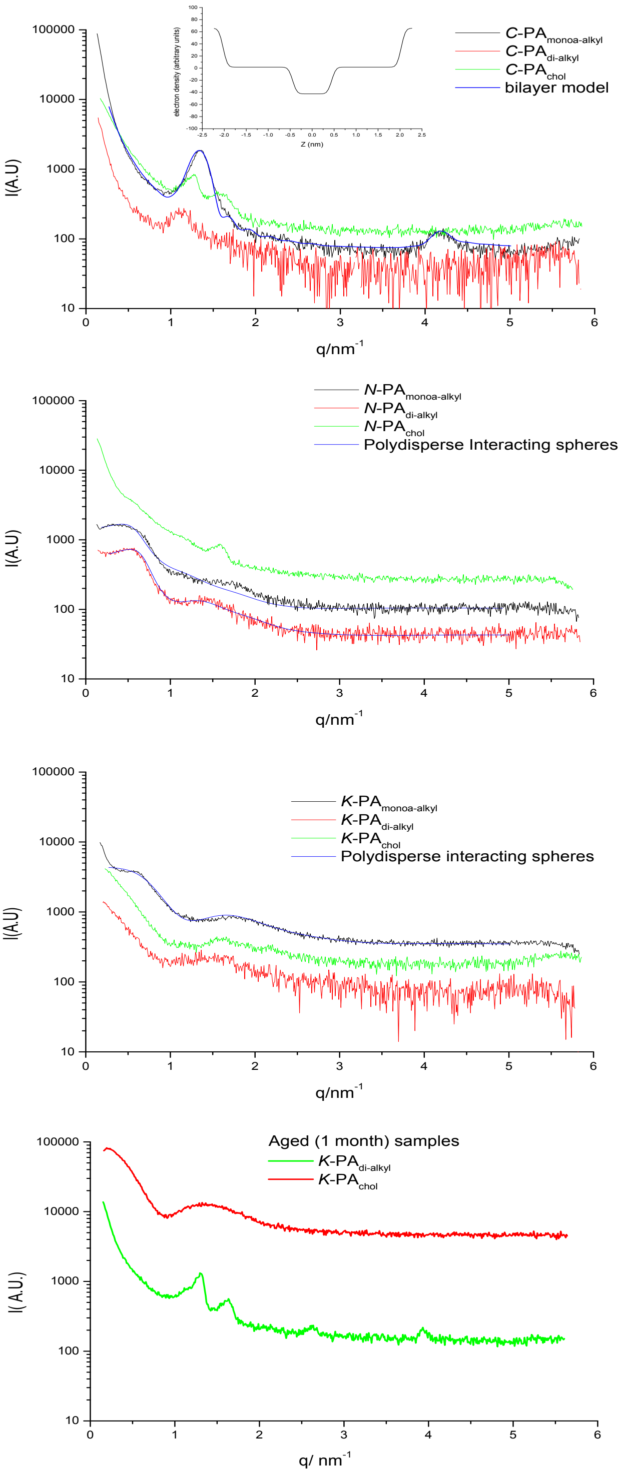

Figure S6. Small Angle X-Ray Scattering as a function of scattering vector modulus measured at 25ºC. Samples were prepared by adding excess water to a PAs pellet and were incubated for 48h at 40ºC. Blue lines correspond to bilayer or interacting polydisperse spheres fits. The parameters of the fits are given in table S4 and the electronic density profile of *C*-$PA_{mono\text{-}alkyl}$ is given as an inset.

FAM-C_{18}

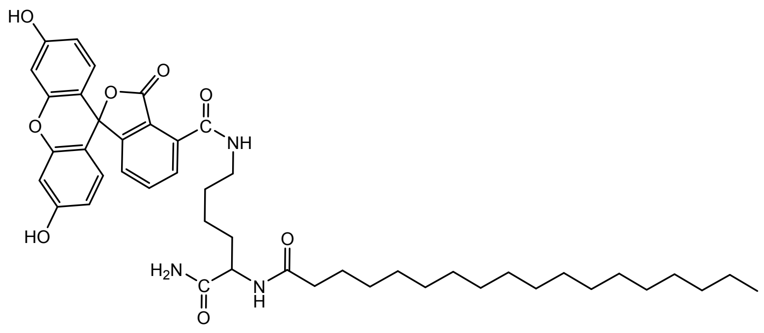

Figure S7. Primary structure of the fluorescent lipophilic derivative FAM-C_{18}

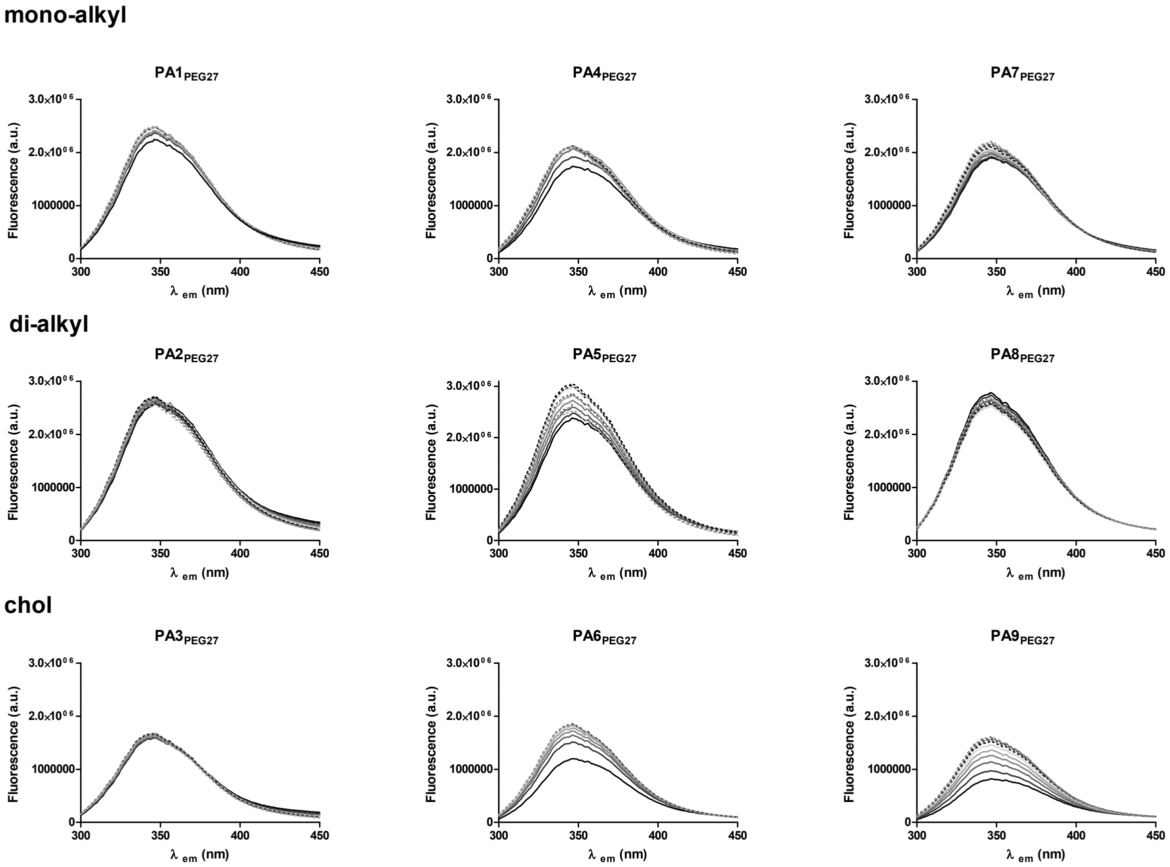

Figure S8. Fluorescence emission spectra of PAs upon titration with POPC unilamellar vesicles. Black solid line represents 5.0 × 10^{-6} M of PA in HEPES buffer (0.01 M, pH 7.4); grey and dotted lines correspond to PA titration with POPC LUVs at a concentration ranged from 12.5 × 10^{-6} M to 2.0 × 10^{-4} M.

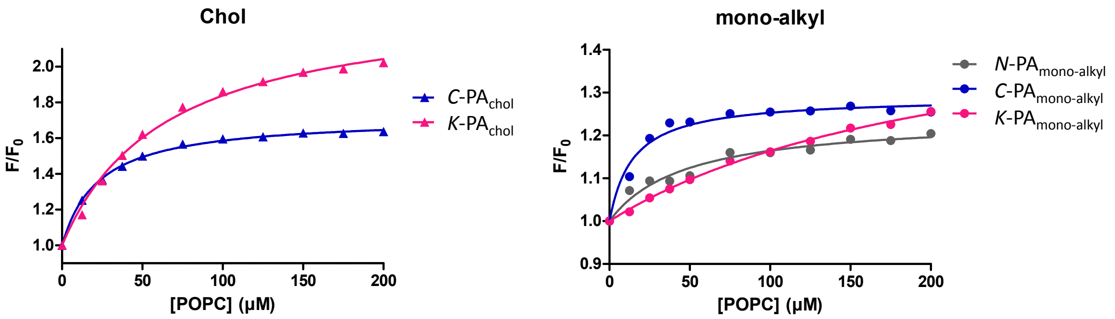

Figure S9. Partitioning isotherms of *C*-PA$_{chol}$ and *K*-PA$_{chol}$ as well as *N*-, *C*-, and *K*-PA$_{mono\text{-}alkyl}$, estimated from the fractional change in Trp fluorescence intensity upon addition of increasing amounts of liposomes. The mole fraction partition coefficients K_x are shown in Table 2.

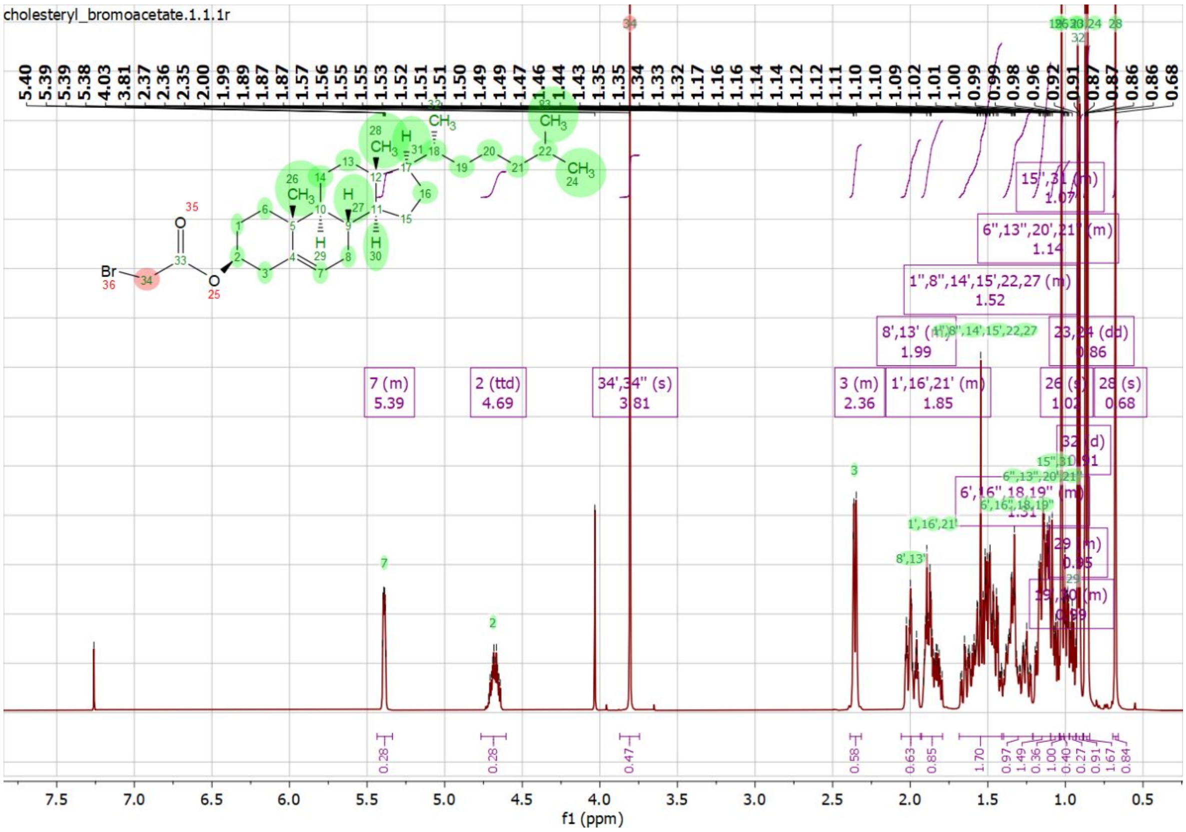
cholesteryl_bromoacetate.1.1.1r
7 (m)
5.39
2 (ttd)
4.69
34',34'' (s)
3.81
3 (m)
2.36
8',13' (m)
1.99
1',16',21' (m)
1.85
1'',8'',14',15',22,27 (m)
1.52
6',16'',18,19'' (m)
1.31
6'',13'',20',21' (m)
1.14
15'',31 (m)
1.07
26 (s)
1.02
32 (d)
0.91
23,24 (dd)
0.86
28 (s)
0.68
Br
f1 (ppm)

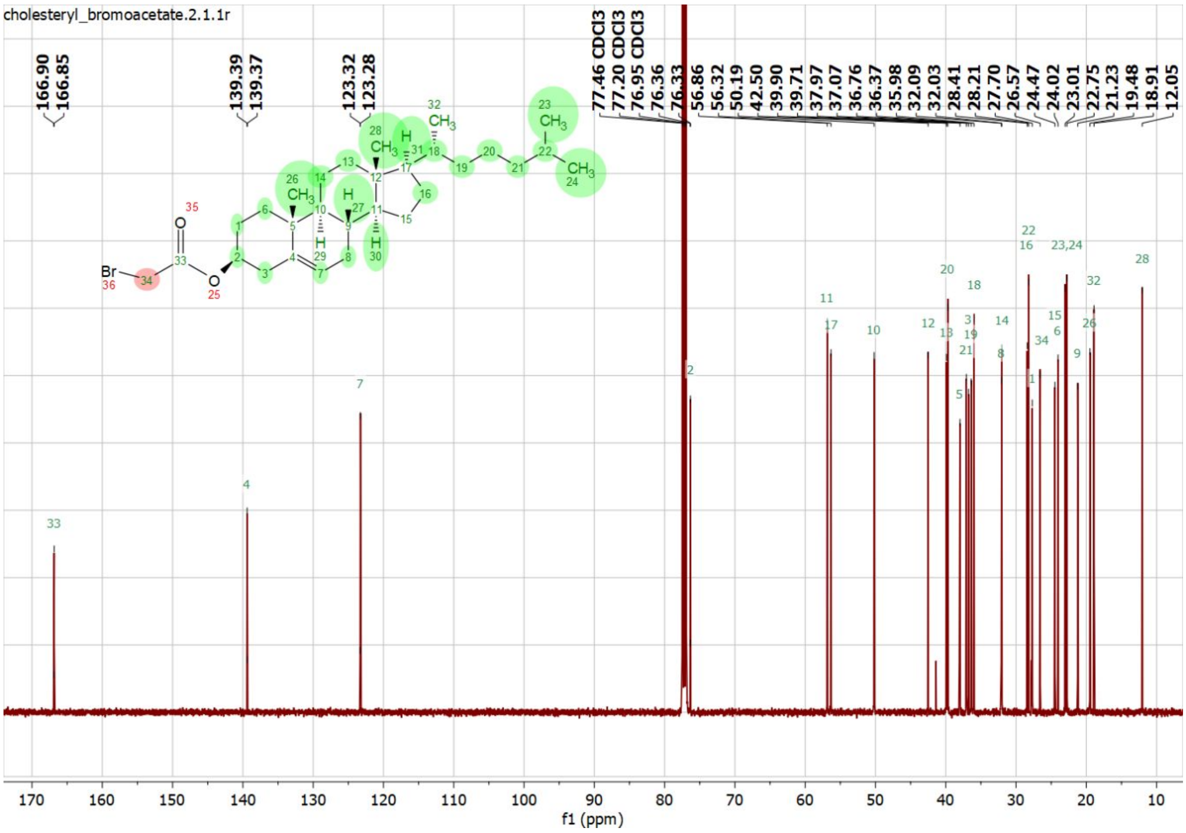
cholesteryl_bromoacetate.2.1.1r
166.90
166.85
139.39
139.37
123.32
123.28
77.46 CDCl3
77.20 CDCl3
76.95 CDCl3
Br
f1 (ppm)

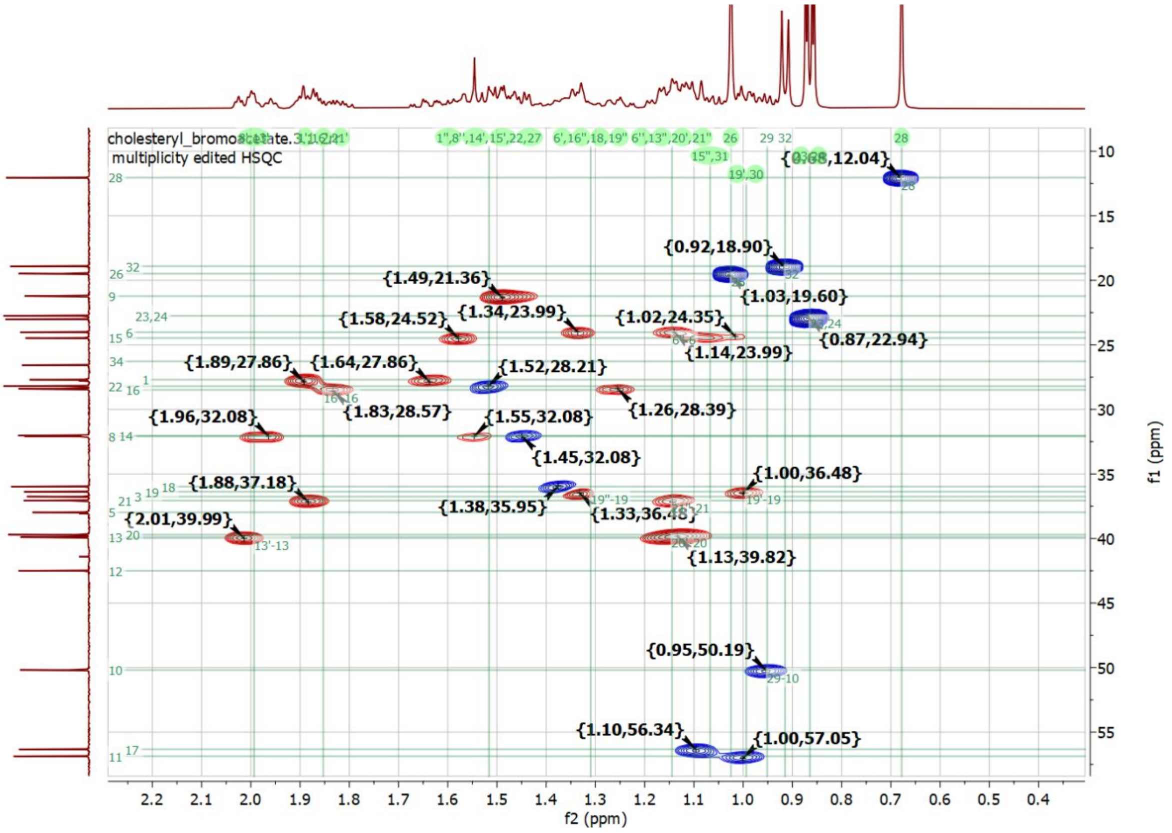

Atom	δ (ppm)
1 C	27.70
H'	1.85
H"	1.52
2 C	76.33
H	4.69
3 C	36.76
H2	2.36
4 C	139.39
5 C	37.97
6 C	24.02
H'	1.31
H"	1.14
7 C	123.28
H	5.39
8 C	32.09
H'	1.99
H"	1.52
9 C	21.23
10 C	50.19
11 C	56.86
12 C	42.50
13 C	39.90
H'	1.99
H"	1.14

Atom	δ (ppm)
14 C	32.03
H'	1.52
H"	1.52
15 C	24.47
H'	1.52
H"	1.07
16 C	28.49
H'	1.85
H"	1.31
17 C	56.32
18 C	35.98
H	1.31
19 C	36.37
H'	0.99
H"	1.31
20 C	39.71
H'	1.14
H"	1.14
21 C	37.07
H'	1.85
H"	1.14
22 C	28.21
H	1.52

Atom	δ (ppm)
23 C	22.75
H3	0.86
24 C	22.75
H3	0.86
25 O	
26 C	19.48
H3	1.02
27 H	1.52
28 C	12.05
H3	0.68
29 H	0.95
30 H	0.99
31 H	1.07
32 C	18.91
H3	0.91
33 C	166.85
34 C	26.28
H'	3.81
H"	3.81

Figure S10. NMR experiments of the cholest-5-en-3-yl bromoacetate.

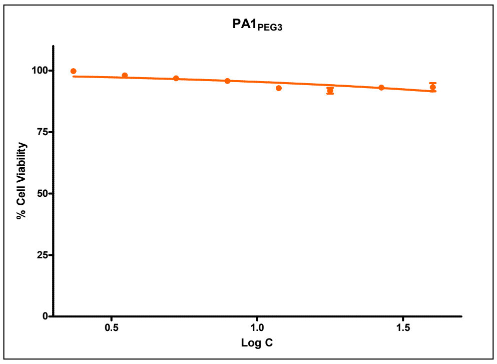
PA1PEG3
100
75
50
25
0
% Cell Viability
0.5
1.0
1.5
Log C

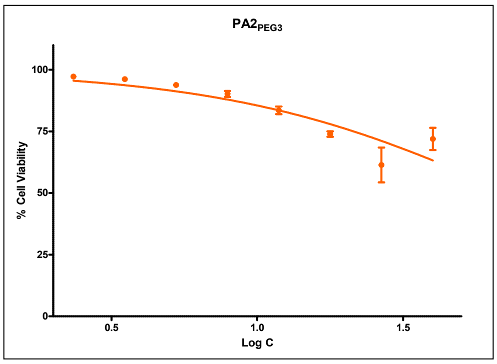
PA2PEG3
100
75
50
25
0
% Cell Viability
0.5
1.0
1.5
Log C

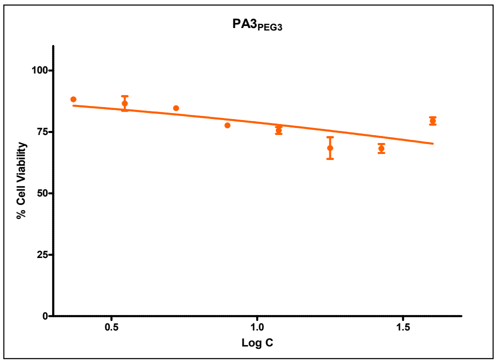
PA3PEG3
100
75
50
25
0
% Cell Viability
0.5
1.0
1.5
Log C

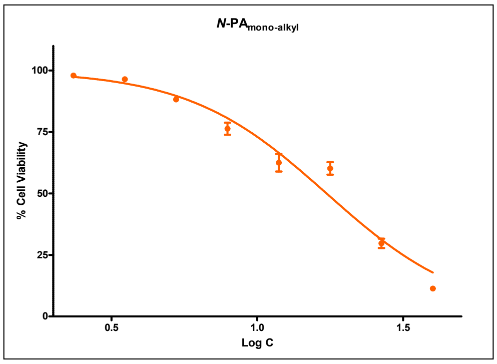
N-PAmono-alkyl
100
75
50
25
0
% Cell Viability
0.5
1.0
1.5
Log C

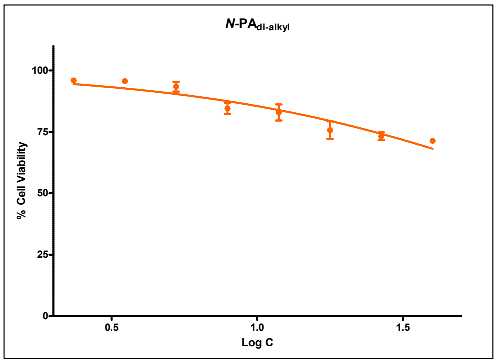
N-PAdi-alkyl
100
75
50
25
0
% Cell Viability
0.5
1.0
1.5
Log C

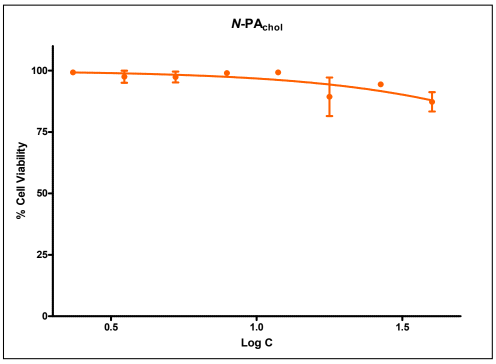
N-PAchol
100
75
50
25
0
% Cell Viability
0.5
1.0
1.5
Log C

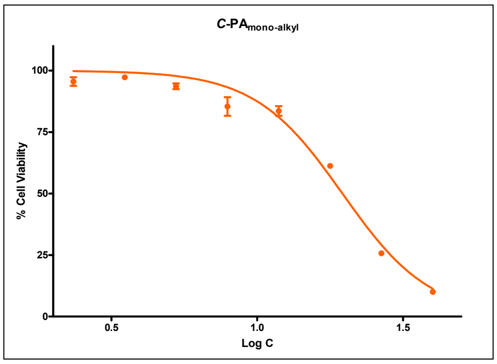
C-PAmono-alkyl
100
75
50
25
0
% Cell Viability
0.5
1.0
1.5
Log C

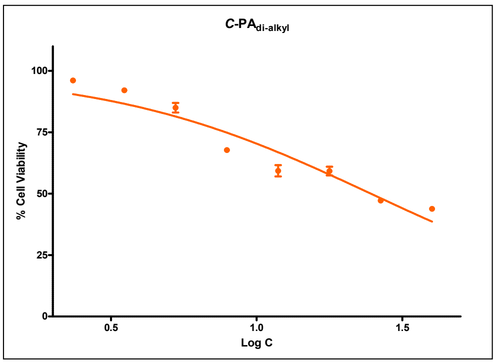
C-PAdi-alkyl
100
75
50
25
0
% Cell Viability
0.5
1.0
1.5
Log C

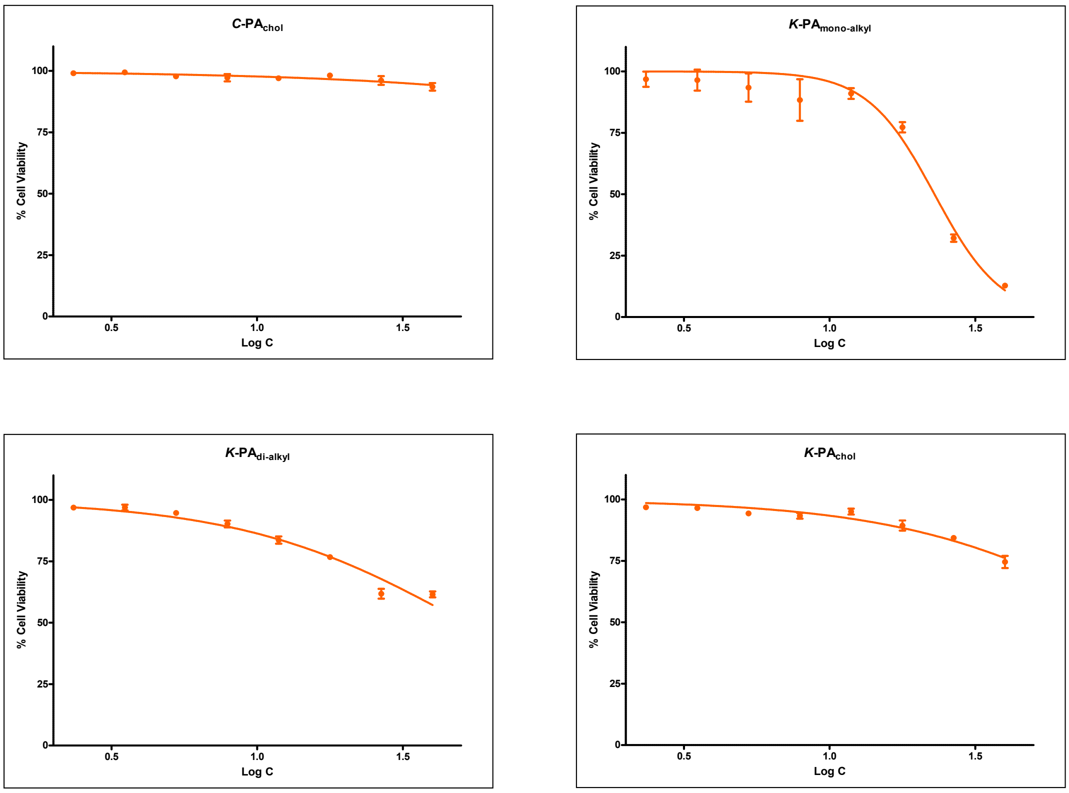

Figure S11. Dose-response curves obtained in MTT cytotoxicity assays.

Table S1. Fitting parameters corresponding to the fits of multilayer model in the form of slabs for C-$PA_{mono\text{-}alkyl}$ **(A)** or polydisperse interacting spheres for *N*-$PA_{mono\text{-}alkyl}$, *N*-$PA_{di\text{-}alkyl}$ and *K*-$PA_{mono\text{-}alkyl}$ **(B)**

(A)

**lamellar**	***C*-$PA_{mono\text{-}alkyl}$**
χ^1	6.4
d^2 (nm)	4.56
η^3	0.0336
N^4	6.0
Z_1 (nm)	0.44
Z_2 (nm)	2.02
ρ_1 ($Z< Z_1$ A.U.)	-42
ρ_2 ($Z_2<Z< Z_1$ A.U.)	1.5
ρ_3 ($d/2<Z< Z_2$ A.U.)	65

(B)

**spheres^2**	***N*-$PA_{mono\text{-}alkyl}$**	***N*-$PA_{di\text{-}alkyl}$**	***K*-$PA_{mono\text{-}alkyl}$**
χ^1	8.4	7.9	8.4
R_1 (nm)	0.15	3.05	1.28
R_2 (nm)	2.60	4.03	3.71
R_3 (nm)	4.75	-	6.50
ρ_1 (A.U.)	212	-13	177
ρ_2 (A.U.)	-54	52	-85
ρ_3 (A.U.)	35	-	19
ϕ^3	0.23	0.28	0.11
R_i^4 (nm)	4.9	7.3	3.6
P.I.^{5}	0.22	0.25	0.48

[1] Reduced chi squared (pure statistical error corresponds to χ=1)
[2] Radii and electron density contrast from the center of the particle to the exterior.
[3] Volume fraction of the hard spheres model.
[4] Effective radii of the hard spheres model.
[5] Polydispersity index

Table S2. Fitting parameters corresponding to the fits of Gaussian models to POPC vesicles and PAs dopped POPC vesicles shown in figure 5A of the main text. The geometrical parameters meaning is given in sheme S1, for more details of the model see references 36 and 37 of the main text.

	***N*-PA$_{mono\text{-}alkyl}$**	***N*-PA$_{di\text{-}alkyl}$**	***N*-PA$_{chol}$**	***K*-PA$_{mono\text{-}alkyl}$**	***K*-PA$_{di\text{-}alkyl}$**	**POPC**
χ[1]	3.5	3.0	0.6	1.4	5.1	3.7
**d[2] (nm)**	7.89	7.79	8.53	7.90	7.81	6.70
η[3]	2.8E-5	3.3E-5	0.19	0.0236	0.0923	0.502
**N[4]**	3.8	3.0	2.5	5.8	25.4	1.6
**N_f[5]**	6.9	7.4	12.2	54.1	35.1	1.0
**σ_h (nm)**	0.252	0.179	0.223	0.277	0.072	0.338
**ρ_h (e/nm^3)**	83	109	137	93	114	110
**Z_h (nm)**	1.84	1.79	1.83	1.82	1.85	1.79
**σ_c (e/nm^3)**	0.494	0.504	0.550	0.368	0.202	0.697

[1] Reduced chi squared (pure statistical error corresponds to χ=1)
[2] Bragg distance corresponding to multilamellar structures.
[3] Caillé parameter (the smaller the more rigid the bilayer).
[4] Number of correlated bilayers.
[5] Number of uncorrelated bilayers

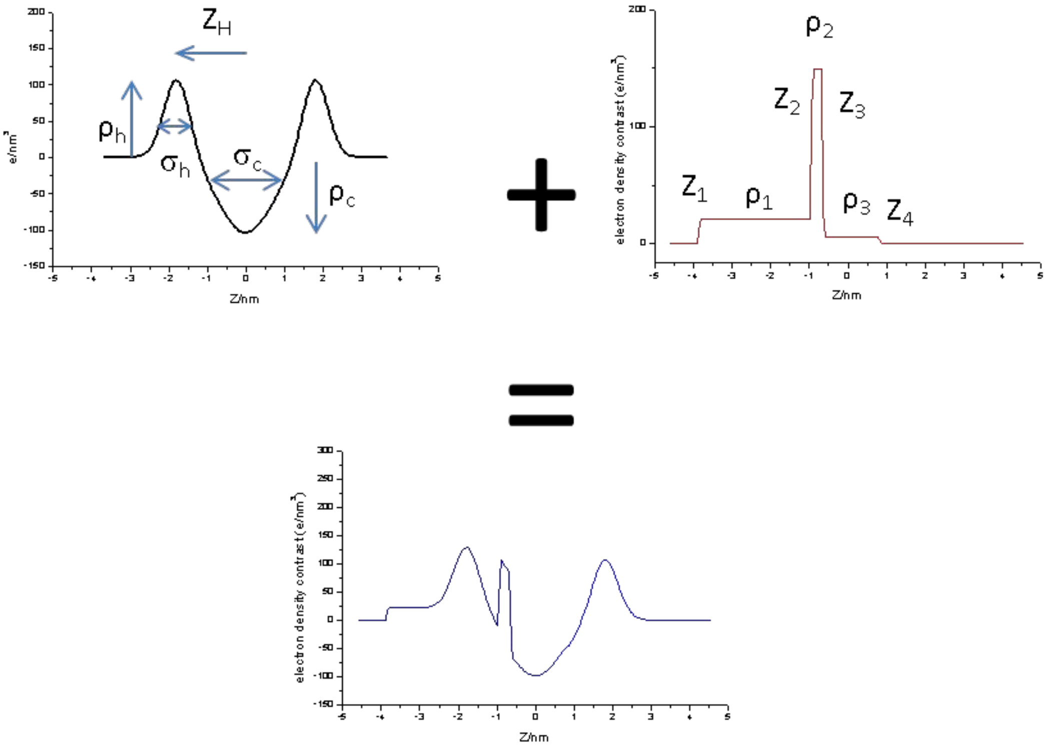

Scheme S1. Description of the geometrical parameters for the symmetric bilayers and the three-slab models.

Table S3. Fitting parameters of the additional slabs for the asymmetric electronic profiles of PAs dopped POPC vesicles corresponding to the fits of Figure 5B of the main text samples. The bilayer parameters used are those of POPC sample in Table S2 (excluding the multilamellarity parameters. The meaning of the slab parameters is given in Scheme S1.

	***C*-PA$_{\text{mono-alkyl}}$**	***C*-PA$_{\text{di-alkyl}}$** *	***C*-PA$_{\text{chol}}$**	***K*-PA$_{\text{chol}}$**
χ	0.9	2.0	2.9	3.2
**Z1 (nm)**	-3.81	-3.88	-4.04	-4.15
**Z2 (nm)**	-0.91	-0.98	-0.93	-1.03
**Z3 (nm)**	-0.73	-0.68	-0.75	-0.83
**Z4 (nm)**	0.71	0.84	0.49	0.19
**ρ_{12} (e/nm^3)**	22	22	9	7
**ρ_{23} (e/nm^3)**	269	144	217	295
**ρ_{34} (e/nm^3)**	6E-4	6	1.7E-11	2.6E-12

* ***C*-PA$_{\text{di-alkyl}}$** shows signs of multilamellarity with the following parameters d=8.33 nm, η=0.029, N=1.81, N_f=5.0

Table S4. Cytotoxic concentration values (CC_{50}) by MTT assay

PA	***CC₅₀*** **(µM)**
**$PA1_{PEG3}$**	> 40
**$PA2_{PEG3}$**	> 40
**$PA3_{PEG3}$**	> 40
***N*-$PA_{mono\text{-}alkyl}$**	17
***N*-$PA_{di\text{-}alkyl}$**	> 40
***N*-PA_{chol}**	> 40
***C*-$PA_{mono\text{-}alkyl}$**	20
***C*-$PA_{di\text{-}alkyl}$**	25
***C*-PA_{chol}**	> 40
***K*-$PA_{mono\text{-}alkyl}$**	23
***K*-$PA_{dialkyl}$**	> 40
***K*-PA_{chol}**	> 40
